# Pan-cancer discovery of somatic mutations from RNA sequencing data

**DOI:** 10.1038/s42003-024-06326-y

**Published:** 2024-05-23

**Authors:** Gongyu Tang, Xinyi Liu, Minsu Cho, Yuanxiang Li, Dan-Ho Tran, Xiaowei Wang

**Affiliations:** 1 Department of Pharmacology and Regenerative Medicine, University of Illinois at Chicago, Chicago, IL USA; 2 Department of Mechanical Engineering and Materials Science, Washington University in St. Louis, St. Louis, MO USA; 3 University of Illinois Cancer Center, Chicago, IL USA

**Keywords:** Software, Cancer genomics

## Abstract

Identification of somatic mutations (**SMs**) is essential for characterizing cancer genomes. While DNA-seq is the prevalent method for identifying SMs, RNA-seq provides an alternative strategy to discover tumor mutations in the transcribed genome. Here, we have developed a machine learning based pipeline to discover SMs based on RNA-seq data (designated as **RNA-SMs**). Subsequently, we have conducted a pan-cancer analysis to systematically identify RNA-SMs from over 8,000 tumors in The Cancer Genome Atlas (**TCGA**). In this way, we have identified over 105,000 novel SMs that had not been reported in previous TCGA studies. These novel SMs have significant clinical implications in designing targeted therapy for improved patient outcomes. Further, we have combined the SMs identified by both RNA-seq and DNA-seq analyses to depict an updated mutational landscape across 32 cancer types. This new online SM atlas, OncoDB (https://oncodb.org), offers a more complete view of gene mutations that underline the development and progression of various cancers.

## Abstract (web summary)

The authors develop a web resource to present pan-cancer mutations in 32 major types of cancer. This new online somatic mutations atlas, OncoDB is publicly available at https://oncodb.org.

## Introduction

The detection of somatic mutations (**SMs**) is critical in characterizing the cancer genome^1^. Historically, SMs were identified using Sanger sequencing or RT-PCR techniques^2^. These low throughput methods were limited to designated sequences, resulting in high experimental costs when examining multiple genomic regions. With the development of next-generation sequencing (**NGS**) technologies, the detection of SMs in the whole exomes or genomes has become much more efficient and cost-friendly using DNA sequencing (**DNA-seq**) methods^3,4^. Currently, researchers commonly employ DNA-seq to identify SMs to investigate the underlying mechanisms of cancer initiation and progression.

RNA sequencing (**RNA-seq**) is another type of NGS approach that is commonly employed for gene expression profiling analysis^5,6^. Since the transcriptome represents a cell’s transcribed genome, it is feasible to detect SMs based on RNA-seq data (designated as **RNA-SMs**) in cancer. In particular, RNA-seq analysis can identify low-frequency mutations in exonic regions that may not be observable in DNA-seq data^7^. To this end, multiple bioinformatics strategies have been developed for detecting SMs based on RNA-seq data. For instance, Sheng et al. identified SMs using a bias-reduced generalized linear model, which was trained on features extracted from RNA-seq data^8^; Yizhak et al. designed an alignment strategy to minimize read errors in RNA-seq data^9^; Muyas et al. developed a machine learning model to distinguish somatic variants from germline variants presented in RNA-seq data^10^.

While these bioinformatics methods have enabled SM detection using RNA-seq data, they had their limitations. Of note, existing methods did not comprehensively consider unique characteristics relevant to RNA-seq data, such as those related to exon splicing, adapter clipping, or RNA editing. Furthermore, most existing methods rely on a single sequence aligner in conjunction with a single variant caller; this strategy could introduce bias that is specifically associated with individual aligners or variant callers, potentially resulting in a relatively high rate of false discoveries. In addition, most existing methods were tailored for the analysis of specific datasets and may not be broadly applied to pan-cancer discovery of RNA-SMs. Thus, there is a strong demand to develop a robust RNA-SM discovery pipeline that can be utilized in diverse types of cancer. To this end, we developed a bioinformatics pipeline, the **I**ntegrated **M**utation **A**nalysis **P**ipeline for **R**NA-seq data (**IMAPR**), for RNA-SM discovery. Importantly, we applied IMAPR to identify RNA-SMs in a pan-cancer cohort of 8048 cases from The Cancer Genome Atlas (**TCGA**). IMAPR identified over 105,000 novel mutations that were not previously discovered in the DNA-seq data from TCGA. Combined analysis of DNA-seq-based somatic mutations (**DNA-SMs**) and RNA-SMs depicted a comprehensive mutational landscape across many cancer types. Based on both TCGA RNA-SMs and DNA-SMs, we developed a web resource, OncoDB, to present pan-cancer mutations in 32 major types of cancer. This newly updated SM atlas offers a more complete view of gene mutations that underline the development and progression of various cancers.

## Results

### Developing a bioinformatics pipeline for RNA-SM detection

**Fig. 1:**
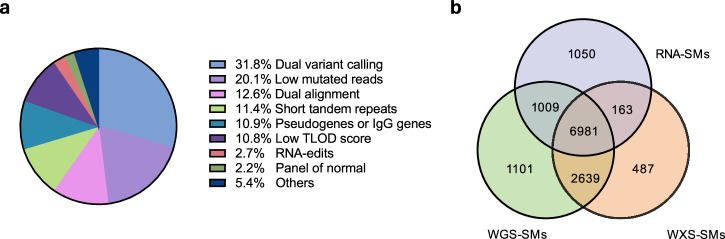
Summary of the mutation filters in the RNA-SM discovery pipeline. **a** Percentage of candidate RNA-SMs rejected by individual mutation filters. **b** Cross-comparison of the RNA-SMs with WXS-SMs and WGS-SMs. The RNA-SMs were identified with 17 mutation filters in the discovery pipeline. The SMs discovered in 100 TCGA tumor samples (45 LUADs, 20 LUSCs, and 35 HNSCs) were included in this analysis.

To develop a robust RNA-SM discovery pipeline, we selected TCGA samples that have all three types of data available, including RNA-seq, whole exome sequencing (**WXS**), and high-coverage whole genome sequencing (**WGS**). This cohort consisted of 45 lung adenocarcinomas (**LUADs**), 20 lung squamous carcinomas (**LUSCs**), and 35 head and neck squamous cell carcinomas (**HNSCs**). First, we employed Mutect2^11^ to call variants using RNA-seq data, and cross-compared the results with DNA-SMs identified from WXS data. Only about 10% of the variants were validated by the WXS data, mainly due to the low accuracy of variant calling from RNA-seq data, as reported previously^9^. To drastically reduce false-positive variants called from RNA-seq data, we developed an RNA-SM discovery pipeline (as outlined in Fig. S1A) by implementing eighteen mutation filters, ten of which were designed specifically for RNA-seq data. These filters significantly reduced the number of false discoveries (Fig. 1a). Details of these filters are presented in Supplementary Methods. Among them, the most prominent ones included the dual variant calling filter (rejecting 31.8% of the candidate variants), low mutated reads filter (rejecting 20.1%), and dual alignment filter (rejecting 12.6%). In this way, we identified 9203 candidate RNA-SMs with sufficient read coverage. Among them, 7144 (77.6%) were validated by the WXS data and 7990 (86.8%) by high-coverage WGS data, respectively (Fig. 1b).

**Fig. 2:**
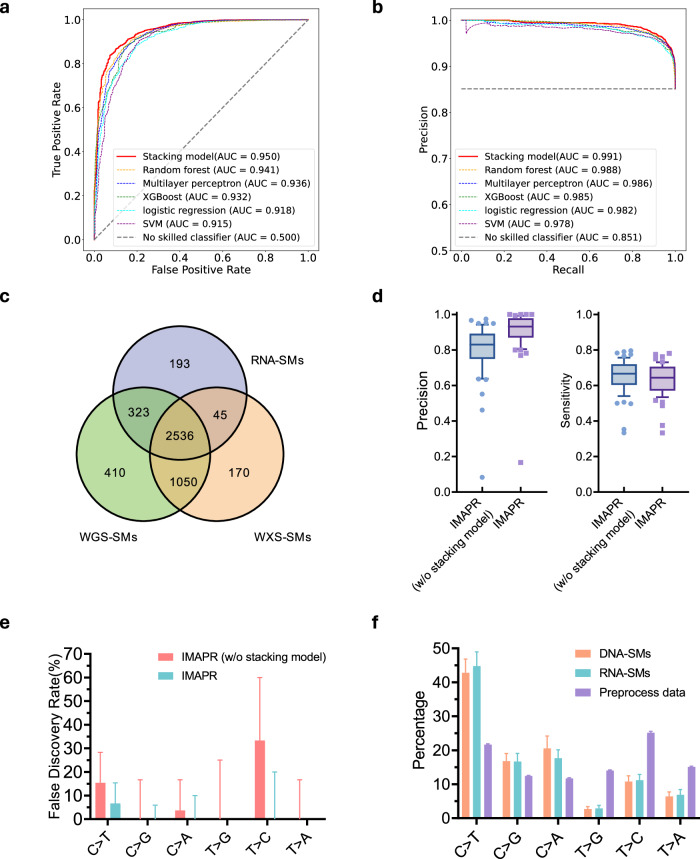
Performance of the IMAPR pipeline as evaluated with an independent validation dataset (including 20 LUSCs and 35 HNSCs from TCGA). Specifically, various machine learning methods were implemented in the pipeline for reducing false discoveries, and their performance was assessed by the AUC values in **a** ROC and **b** PR curve analyses. **c** Cross-comparison of the RNA-SMs identified by IMAPR with WXS-SMs and WGS-SMs in the validation dataset. **d** Impact of the stacking learning model on IMAPR performance. Precision and sensitivity distributions of the RNA-SMs across individual tumors as discovered by IMAPR, with or without the stacking model. Median, 10th and 90th percentiles are displayed in the boxplots. **e** Average false discovery rate (FDR) of the RNA-SMs for each type of allele substitution across tumors. The impact of the stacking model on the FDR of individual substitution types was assessed. **f** Average percentages of allele substitution for base transition types across tumors by individual prediction algorithms. The DNA-SMs and RNA-SMs, identified by Mutec2 and IMAPR, respectively, were compared for base transition distributions. Preprocessed RNA-SM candidates (before applying IMAPR) are also presented as references.

In our analysis, we found that 11.4% (1050/9203) of the RNA-SMs were not validated by either the WXS or WGS data. These variants could have been generated through RNA-specific processes, such as RNA-editing events not recorded in existing databases. To further improve the detection of RNA-SMs, we developed a machine learning approach to distinguish between RNA-only mutations and those present in both DNA- and RNA-seq data. Using our training dataset comprised of RNA-SMs from 45 LUAD samples, we employed five classification-based machine learning methods (detailed in Methods) and evaluated the performance of each one using receiver operating characteristic curve (**ROC**) and precision-recall (**PR**) curves (Fig. S2a, b). Based on these curves, three top-performing learning methods, including random forest, XGboost, and multiplayer perceptron, were further assembled to build a Stacking model based on logistic regression (Fig. S2c). To validate the general applicability of the Stacking model, we applied it to an independent validation dataset consisting of the RNA-SMs from 20 LUSC and 35 HNSCC samples. The Stacking model achieved the best performance, with an ROC-AUC of 0.950 and PR-AUC of 0.991 (Fig. 2a, b). This led to a drastically reduced portion of RNA-only mutations from 14.9% (521/3503) to 6.2% (193/3097) in the validation cohort (Fig. 2c). Furthermore, median precision of RNA-SM detection for the patients improved from 0.831 to 0.932, while retaining a sensitivity of 0.650 (Fig. 2d). Thus, we integrated this Stacking machine learning approach with aforementioned RNA-SM calling process to create IMAPR, a bioinformatics pipeline for RNA-SM discovery.

Numerous studies indicate that RNA editing is an important factor contributing to falsely discovered variants^12^. Deamination of adenosine is the most common form of RNA editing, resulting in T > C transitions as observed in RNA-seq data^13^. To minimize the impact of RNA editing events, we applied an RNA editing filter to detect all known RNA editing sites as well as the aforementioned Stacking model to predict previously unidentified ones. In order to evaluate the effectiveness of the Stacking model, we calculated the false discovery rates (**FDRs**) for all transition types (Fig. 2e). Our results showed that, without the Stacking model, the FDR for T- > C transition remained relatively high, likely due to the misidentification of RNA editing sites as RNA-SMs. In contrast, the Stacking model significantly reduced the FDR for RNA-SMs across all transition types. Furthermore, we compared the percentages of allele substitution for each transition type among the RNA-SMs and DNA-SMs. Our analysis revealed that preprocessed data had a higher percentage of T > C transitions compared to DNA-SMs (Fig. 2f). Our RNA-SM discovery pipeline significantly reduced the number of false discoveries with T > C transitions, resulting in a similar percentage distribution for allele substitutions compared with the DNA-SMs. In summary, IMAPR can effectively filter out RNA editing events to reduce false discoveries in RNA-SM analysis.

Furthermore, we performed a comparative analysis using the aforementioned TCGA validation dataset. Specifically, we evaluated the performance of IMAPR and two existing tools, with the F-score and ROC-AUC metrics presented in Fig. S3a, b. The F-score analysis demonstrated that IMAPR has the best performance, achieving a score of 0.372, compared with 0.317 and 0.339 for RNA-Mutect and RNA-SSNV, respectively. Similarly, in the ROC analysis, IMAPR showed improved performance with an AUC of 0.950 (vs. AUC = 0.913 for RNA-SSNV). As for RNA-Mutect, it employs a filter-based classification method and does not compute probabilistic scores; thus, only one datapoint was recorded in the ROC figure, with true positive rate and false positive rate to be 0.844 and 0.224, respectively. In summary, both F-score and ROC analyses demonstrated improved performance of IMAPR over existing methods. To further ascertain IMAPR’s general applicability, we also applied the IMAPR pipeline to independent data (the Mun dataset)^14^. In this analysis, 81.4% (2016/2476) of the mutations identified via RNA-seq data could be confirmed by the corresponding WXS data. Our analysis revealed a median precision of 0.714 and a recall of 0.640 across all 80 patients in this dataset (Fig. S3c). Additionally, the IMAPR pipeline achieved an ROC-AUC of 0.828 (Fig. S3d). These results on the Mun dataset aligned well with our TCGA analysis, in which we tested and validated the IMAPR pipeline using TCGA WXS data as a reference, achieving similar results (precision: 0.712; recall: 0.638; ROC-AUC: 0.825).

### RNA-SM validation by high-coverage WGS data

**Fig. 3:**
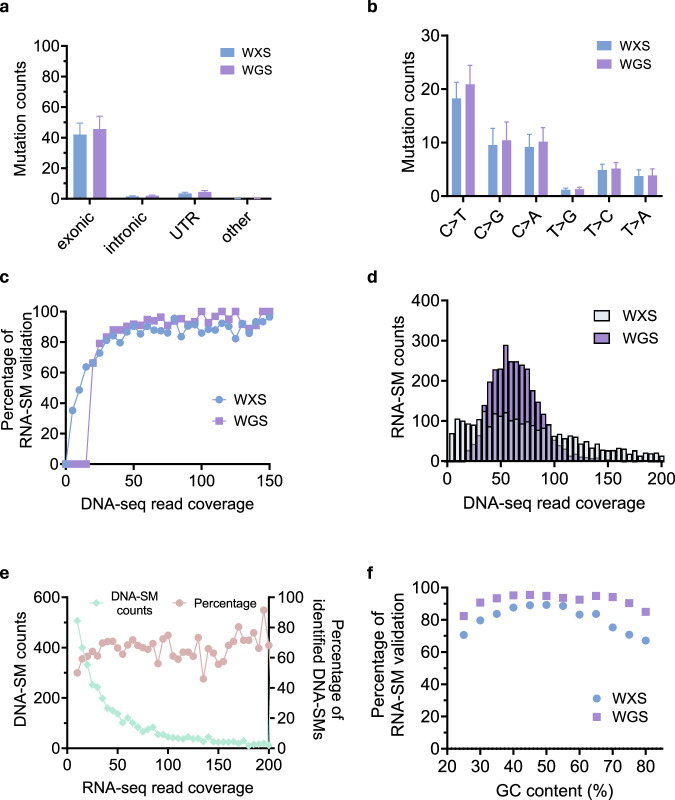
Using TCGA WGS and WXS data as validation reference for RNA-SM analysis. **a** Distributions of RNA-SM counts in different genic regions across the tumors as validated by the WXS/WGS data. **b** Distribution of RNA-SM counts for each type of allele substitution across the tumors as validated by the WXS/WGS data. **c** Percentage of validated RNA-SMs among all predicted ones in relation to read depth of the mutation sites. Specifically, the RNA-SMs were validated by TCGA WXS or WGS data. **d** DNA sequencing coverage of the RNA-SM sites in the WXS or WGS data. **e** Overlap of the DNA-SMs with the RNA-SMs. The DNA-SM counts in relation to RNA-seq read depth is presented on the left y-axis; the percentage of the DNA-SMs that overlapped with the RNA-SMs is presented on the right y-axis. **f** Percentage of validated RNA-SMs in relation to the GC content of the mutation sites. The GC content was determined from a 100 bp window surrounding a mutation site.

In our TCGA validation dataset, which included 20 LUSC and 35 HNSC samples, we found that the validation rate of RNA-SMs was higher with high-coverage WGS data (92.3%, 2859/3097) than WXS data (83.3%, 2581/3097, Fig. 2c). To identify potential reasons for the difference in the validation rates, we compared the RNA-SMs validated by the WXS and WGS data, respectively. First, our analysis indicated that both WGS and WXS data provided consistent validation results in terms of genic regions and allele substitutions of the RNA-SMs. Specifically, most RNA-SMs validated by the WXS data were located in exonic and UTR regions, as were most RNA-SMs validated by the WGS data (Fig. 3a). Moreover, WGS-validated RNA-SMs and WXS-validated RNA-SMs exhibited similar characteristics of allele substitutions, with a higher percentage of C > T substitution (Fig. 3b).

Next, we further investigated the relationship between the DNA-seq coverage and the number of validated RNA-SMs. The result indicated that deeper read depth in the DNA-seq data was associated with a higher percentage of validated RNA-SMs. In both WGS and WXS data, over 80% of RNA-SMs were validated when the read depth of an RNA-SM site was >50 (Fig. 3c). To better understand the impact of sequencing coverage on the validation rate of RNA-SMs, we determined the read depth distributions for the RNA-SM sites in the DNA-seq data. (Fig. 3d). We found the read depth of the WGS data was significantly deeper (72 ± 24) than the WXS data (60 ± 72; Student’s *t*-test, *p* value <0.001); this suggests that deeper read depth of mutation sites in the WGS data led to improved validation rate for the RNA-SMs. We also determined the coverage distribution of the DNA-SM sites in the RNA-seq data (Fig. 3e). Many DNA-SM sites had fewer than 30 reads represented in RNA-seq data, and only ~50% of these DNA-SMs were discovered using the RNA-seq data. In contrast, when the RNA-seq depth of the DNA-SM sites exceeded 50, ~70% of the DNA-SMs were discovered. This suggested that insufficient RNA-seq coverage was the main reason why certain SMs were missed. Thus, sufficient sequencing coverage was an important factor for detecting or validating SMs.

Further, it has been reported that WXS is ineffective in capturing sequences with high or low GC content^15,16^. To investigate the relationship between the GC content and the validation rate of RNA-SMs, we calculated the GC content of 100 neighboring nucleotides surrounding each RNA-SM site. We found that RNA-SMs with extreme GC content (≥70% or ≤30%) were more likely to be validated with the WGS data (~85%) than the WXS data (~70%) (Fig. 3f). Thus, high-coverage WGS data provided the best reference for validating identified RNA-SMs.

### Independent validation of the RNA-SMs in cervical cancer

**Fig. 4:**
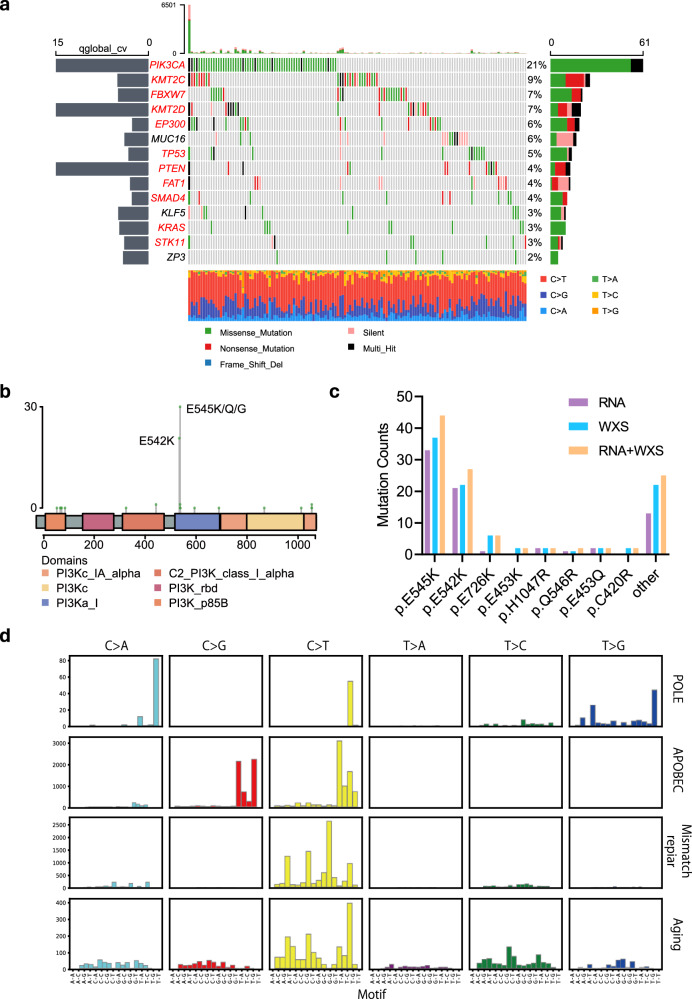
The RNA-SM profile of TCGA cervical cancer. **a** The SMGs in cervical cancer, as identified with the RNA-SMs using dNdScv. Genes labeled in red were also identified from the DNA-SM analysis. **b** Counts of the RNA-SMs in *PIK3CA* across different protein domains. The positions of amino acids are displayed along the x-axis. **c** Counts of the SMs in *PIK3CA* that led to amino acid substitutions, as identified with the RNA-seq, WXS, and combined data. **d** Mutational signatures in cervical cancer, as identified from the RNA-SM data. These included DNA mismatch repair, APOBEC, Aging, and POLE.

To further evaluate the performance of our RNA-SM discovery pipeline, we applied it to detect SMs using independent RNA-seq data from cervical squamous cell carcinomas (**CESCs**) in TCGA. A total of 29,237 RNA-SMs were detected in 297 CESC cases. There is a lack of WGS data for CESCs in TCGA; thus, we used the DNA-SMs identified from the WXS data for comparative analysis with the RNA-SMs. Our analysis revealed that 71.3% of the RNA-SMs overlapped with the DNA-SMs in CESCs; in addition, 8379 novel SMs were identified from the RNA-seq data. Next, we conducted significantly mutated gene (**SMG**) analysis for the RNA-SMs and identified 14 mutated genes (adjusted *p* value <0.05, Fig. 4a). Of these, *PIK3CA*, *KMT2C*, *FBXW7*, *EP300*, *KMT2D*, *PTEN*, *TP53*, *SMAD4*, *KRAS*, *STK11*, and *FAT1* were also found to be significantly mutated by DNA-SM analysis (Fig. S4). Additionally, RNA-SMs led to the identification of three new SMGs, including *KLF5*, *MUC16*, and *ZP3*. Previous studies linked MUC16 mutations to an immune response in cervical cancer^17^, while *KLF5* was associated with tumorigenesis in cervical tissues^18^, and *ZP3* has been identified as a potential target for immunotherapy in ovarian cancer^19^.

*PIK3CA* was the most frequently mutated SMGs in cervical cancer, as revealed by both RNA-seq and DNA-seq analyses (RNA-seq: 21% of all available CESCs; DNA-seq: 28%; Fig. 4a and Fig. S4). Interestingly, combined analysis of the RNA-SMs and DNA-SMs led to improved sensitivity for detecting *PIK3CA* mutations (31%, Fig. S5). To further characterize the expanded pool of SMs, we conducted an amino acid replacement analysis to annotate the mutations at the protein level. RNA-SM analysis revealed E542K and E545K as the most abundant *PIK3CA* mutations (Fig. 4b), which was consistent with the findings from DNA-SM analysis (Fig. S6). In particular, for mutations detected in RNA-seq data only, most of them were E542K and E545K rather than the ones residing in other *PIK3CA* subunits (Fig. 4c). Notably, these mutations were found in the catalytic subunit p110α, leading to a gain of function to activate AKT signaling and induce oncogenic transformation in cervical cancer^20^. Overall, these findings suggested that the identification of RNA-SMs could contribute to a more comprehensive characterization of the cancer genomes.

Furthermore, mutational signature analysis was performed to determine the etiology of cervical cancer using the Signatureanalyzer package^21^. DNA mismatch repair, APOBEC, aging, and polymerase epsilon (**POLE**) were identified as the most significant RNA-SM signatures (Fig. 4d). When compared with DNA-SM mutational signatures (Fig. S7), RNA-SM analysis detected consistent signatures, including APOBEC, aging, and DNA mismatch repair. Notably, a novel signature, POLE, was identified with the RNA-SM data. Previous studies have linked POLE mutations to the development of multiple cancers, due to the loss of proofreading function resulting in accumulation of mutant genes in cells^22^. These results suggested that both RNA-seq and DNA-seq data were useful for the discovery of SMs in cervical cancer. Additionally, RNA-seq-based mutation analysis identified novel SMs that were missed by DNA-seq analysis alone.

### Pan-cancer RNA-SM profiles in the TCGA cohort

We employed the IMAPR pipeline to analyze the RNA-seq data from 32 cancer types in the TCGA dataset. The cancer types included in this study are adrenocortical carcinoma (**ACC**), bladder urothelial carcinoma (**BLCA**), brain lower grade glioma (**LGG**), breast invasive carcinoma (**BRCA**), CESC, cholangiocarcinoma (**CHOL**), colon adenocarcinoma (**COAD**), esophageal carcinoma (**ESCA**), glioblastoma multiforme (**GBM**), HNSC, kidney chromophobe (**KICH**), kidney renal clear cell carcinoma (**KIRC**), kidney renal papillary cell carcinoma (**KIRP**), liver hepatocellular carcinoma (**LIHC**), LUAD, LUSC, lymphoid neoplasm diffuse large B-cell lymphoma (**DLBC**), mesothelioma (**MESO**), ovarian serous cystadenocarcinoma (**OV**), pancreatic adenocarcinoma (**PAAD**), Pheochromocytoma and Paraganglioma (**PCPG**), Prostate adenocarcinoma (**PRAD**), rectum adenocarcinoma (**READ**), sarcoma (**SARC**), skin cutaneous melanoma (**SKCM**), stomach adenocarcinoma (**STAD**), testicular germ cell tumors (**TGCT**), thymoma (**THYM**), thyroid carcinoma (**THCA**), uterine carcinosarcoma (**UCS**), uterine corpus endometrial carcinoma (**UCEC**), and uveal melanoma (**UVM**).

**Fig. 5:**
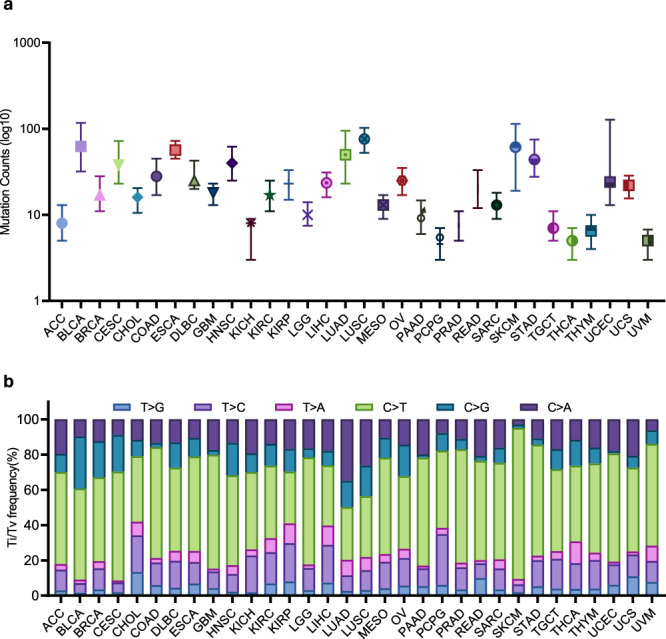
Pan-cancer RNA-SM profiles in TCGA. **a** Distribution of RNA-SM counts in individual patients across 32 types of cancer. **b** Composition of allele transition frequency in RNA-SMs across 32 types of cancer.

Our analysis revealed that LUSC, BLCA, SKCM, and LUAD had the highest numbers of mutations, in contrast to the lowest numbers for PCPG, THCA, and UVM (Fig. 5a). Comparison of mutational spectra across 32 types of cancer revealed the largest difference being C > T transitions and C > G transversions (Fig. 5b). Of note, our analysis found the highest C > T transition rate in SKCM (~85% of total SMs), consistent with previous findings linking C > T transitions to skin cancers caused by ultraviolet light exposure^23^. In addition, we also observed a high rate of C > A transversion in LUAD and LUSC, which has been associated with tobacco smoking^24^.

Interestingly, we observed a strong positive correlation between the RNA-SM and DNA-SM counts across all cancer types, indicating consistent findings across RNA-seq and DNA-seq analyses (Fig. S8a). Further, we characterized the somatic mutations in 32 cancer types from both DNA-seq and RNA-seq data (Table 1). Missense mutations are the predominant type found in both DNA- and RNA-seq data. As for other mutation types, fewer indels but more missense and silent mutations are detected in the RNA-seq data compared with DNA-seq data. Importantly, we discovered novel RNA-SMs that were not previously reported from TCGA DNA-seq analysis. Thus, combined analysis of DNA-SMs and RNA-SMs presented a more comprehensive mutational landscape across individual cancer types (Fig. S8b).

**Table 1:** Comparison of somatic mutation types detected in DNA-seq and RNA-seq data

	DNA-SMs	RNA-SMs
Missense mutation	61.7%	66.5%
Nonsense mutation	5.0%	3.1%
Silent mutation	23.3%	27.4%
Indel	5.3%	1.5%
Others	4.6%	1.4%

### Characteristics of the RNA-SM profiles for key tumor suppressors and oncogenes

**Fig. 6:**
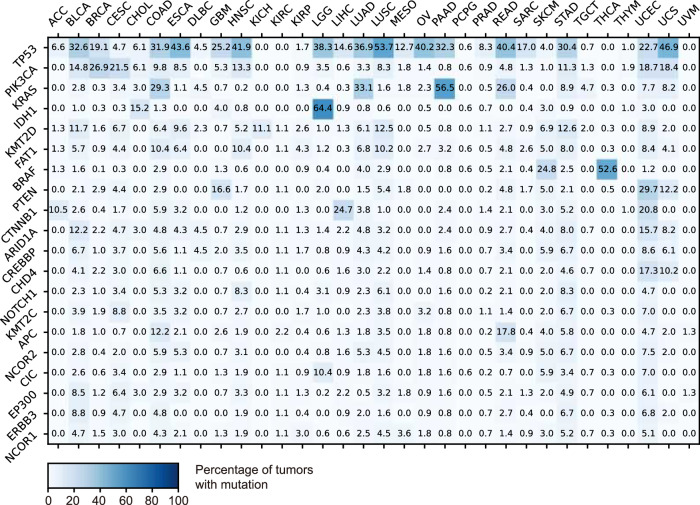
Percentage of the tumors that harbor RNA-SMs in selected oncogenes/tumor suppressors across 32 cancer types. The top 20 most mutated genes are presented. The list of oncogenes/tumor suppressors was obtained from the COSMIC database.

We obtained a set of 197 hallmark tumor suppressors and oncogenes from the Catalog of Somatic Mutations in Cancer (**COSMIC**)^25^ database and analyzed their mutation rates across 32 types of cancer by RNA-SM analysis. Twenty-five most frequently mutated genes across all cancer types are presented in Fig. 6. Our analysis revealed that *TP53* was the most frequently mutated gene in the pan-cancer cohort (23.2%). In twelve types of cancer (including BLCA, COAD, ESCA, HNSC, LGG, LUAD, LUSC, OV, PAAD, READ, STAD, and UCS), *TP53* mutations were observed in over 30% of all cases. Notably, we observed that multiple genes belonging to the same pathways were frequently mutated in various cancers. For instance, the PI3K signaling pathway has been reported to be frequently over-activated in a variety of cancer types^26^. In particular, mutations in *PIK3CA* were frequently observed in BRCA, CESC, UCEC, and UCS, whereas *PTEN* mutations were commonly identified in GBM and UCEC. As another example, alterations in histone modifications can also contribute to the development of cancer by impacting genome stability and gene expression^27^. Our results revealed frequent mutations in histone modification genes across many cancer types. In particular, mutations in *KMT2D*, *KMT2C*, and *ARID1A* were frequently observed in BLCA, STAD, and UCEC.

Besides commonly shared gene mutations, our analysis also revealed mutational profiles that were uniquely associated with specific cancer types. For instance, SKCM had specific mutations in *BRAF* that were driven by ultraviolet light exposure; *IDH1* mutation, which was reported as molecular markers in gliomas^28^, were uniquely identified in LGG; *APC* was frequently mutated in colorectal cancers (COAD and READ), which was considered a key factor in colorectal tumorigenesis^29^; *CTNNB1* in the Wnt/β-catenin pathway was highly mutated in LIHC.

Furthermore, we compared the top 10 or top 40 most mutated genes in 32 types of cancer, as detected by RNA-seq and DNA-seq analysis, respectively (Fig. S9a, b). Most top-ranking mutations were identified from both RNA-seq and DNA-seq data. Additionally, we generated updated mutation profiles for oncogenes and tumor suppressors in various cancers by combining the RNA-SMs and DNA-SMs (Fig. S9c). Of note, the updated mutation profiles included the SMs detected specifically in the RNA-seq data but not in the DNA-seq data, resulting in an expanded view of mutations in cancer patients. For example, *KRAS* was reported to play a key role in malignancy in non-small cell lung cancer^30^. In our analysis, 26.8% of the patients in LUAD were identified with *KRAS* mutations using the WXS data, while 32.0% were identified with *KRAS* mutations using combined RNA- and DNA-seq data. Thus, alterations in these cancer genes were likely to be functionally involved in a larger patient cohort than previously appreciated.

Beyond key tumor suppressors and oncogenes, we performed additional analysis and systematically identified a set of highly mutated genes at the transcriptome level. The mutation frequency of these genes across 32 types of cancer is presented in Fig. S10. *TP53* still emerged as the most mutated gene across various cancer types. Interestingly, we also observed high mutation rates in multiple novel genes, such as *TUBA1C*, *MICB*, and *UQCRHL*. These may represent new mutations only discovered from the RNA-seq data, but not the DNA-seq data. Further functional studies are needed to validate and characterize these novel mutations.

### Mutational signature analysis using the RNA-SMs

**Fig. 7:**
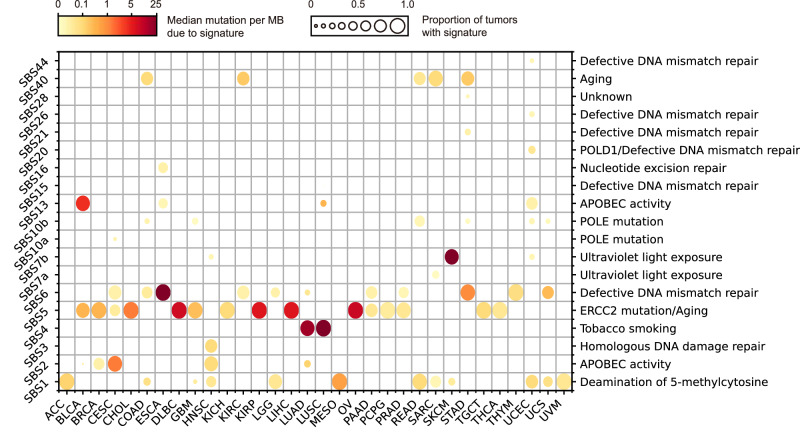
Pan-cancer mutational signatures discovered using the RNA-SMs across 32 cancer types. The size of each dot represents the proportion of tumors with the mutational signature in each cancer type. The color of each dot represents median mutational burden in all individual tumors of the same type. Only tumors with identified mutational signature were included in the analysis.

We conducted a mutational signature analysis using identified RNA-SMs to predict the etiology of each type of cancer. Interestingly, several common endogenous mutational signatures, including DNA deamination, DNA mismatch repair, and aging, were present in most types of cancer (Fig. 7). In addition, we identified cancer type-specific mutational signatures. For example, smoking was found to be the primary cause of lung cancer (LUAD and LUSC); UV exposure was closely associated with SKCM; APOBEC activity, which was reported to be highly correlated with viral infection^31^, contributed to virus-induced cancers, including CESC, HNSC, BLCA, and UCEC. These findings suggest that mutational signatures determined from the RNA-SMs can provide valuable insights into the underlying mechanisms in cancer development. Furthermore, we combined the RNA-SMs and DNA-SMs to depict a more comprehensive profile of the mutational signatures in cancer patients (Fig. S11).

### Clinical implications for cancer patients with novel RNA-SMs

**Fig. 8:**
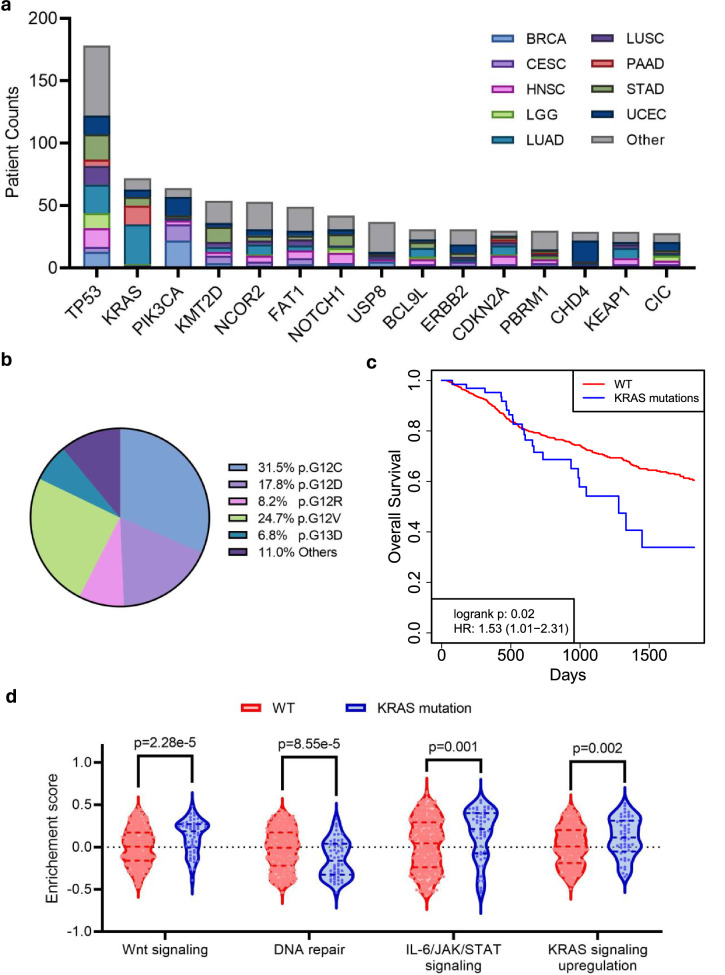
Patient mutations uniquely identified by RNA-SM analysis. **a** Number of patients carrying specific gene mutations as identified by RNA-SM analysis exclusively. Top 15 genes with most mutations are displayed in the graph. Additionally, for each gene, the distribution of patients across cancer types is also presented. **b**–**d** *KRAS* mutations, uniquely identified by RNA-SM analysis, were presented as an example. **b** Composition of *KRAS* mutations among cancer patients. **c** Kaplan–Meier survival analysis to evaluate the prognostic significance of *KRAS* mutations. Patients with wild-type *KRAS* were included as a control group. **d** Selected MSigDB hallmark pathways associated with *KRAS* mutations. Enrichment scores calculated by GSVA were presented.

To evaluate the clinical implications of the RNA-SMs, we focused on novel SMs that were exclusively identified from RNA-SM analysis (i.e., novel mutations missed by DNA-SM analysis). Top 15 hallmark tumor suppressors and oncogenes with most mutations are presented in Fig. 8a. Additionally, for each gene, the distribution of patients across cancer types is also presented. Among these genes, *TP53* was most frequently mutated across 32 cancer types. Cox regression analysis showed that patients with *TP53* mutations demonstrated worse overall survival compared to those without such mutations (*p* value = 7.78E-4, HR = 1.61, 95% CI: 1.22–2.12).

Moreover, we identified many novel mutations in the *KRAS* gene, previously known for being significantly mutated in adenocarcinoma^32,33^. Our RNA-SM analysis showed these novel mutations predominantly occur in various forms of adenocarcinoma, including LUAD, PAAD, and STAD. We also discovered that newly identified mutations in *KRAS* were mainly located in the 12th or 13th codons (Fig. 8b). The presence of these *KRAS* mutations is associated with inferior survival outcomes (*p* value = 0.02, HR = 1.53, 95% CI: 1.01–2.31, Fig. 8c). Lastly, gene set variation analysis highlighted specific pathways that were regulated by these mutations (Fig. 8d). Interestingly, we found that WNT signaling, IL-6/JAK/STAT signaling, and *KRAS* signaling activities were elevated in patients with *KRAS* mutations, in contrast to decreased activity of the DNA repair pathway. Besides *TP53* and *KRAS*, we also discovered many novel mutations in *PIK3CA* and *CDH4*. The occurrence of *PIK3CA* and *CDH4* mutations were linked to better survival outcomes (*PIK3CA*: *p* value = 0.02, HR = 0.42, 95% CI: 0.20–0.88; *CDH4*: *p* value = 0.04, HR = 0.24, 95% CI: 0.05–0.95). In summary, RNA-SM analysis provided valuable mechanistic insights into novel gene mutations that are implicated in cancer outcomes and therapeutic development.

## Discussion

Sequencing coverage is a critical factor in detecting SMs from both DNA-seq and RNA-seq data. Higher coverage results in more accurate SM detection, especially for low-frequency ones. In RNA-seq data, highly expressed genes generally have more reads, which boosts the power of detecting SMs in these exonic regions. However, SM discovery using RNA-seq data faces three major challenges: (1) errors introduced by reverse transcription (**RT**) during sequencing library construction, (2) increased alignment errors near splicing junctions, and (3) RNA editing sites that may be misidentified as SMs. In particular, most false discoveries due to RT errors are associated with low allele frequency or poor sequencing quality. Various variant callers implemented different strategies for detecting low-frequency variants, as well as distinct criteria for filtering sequencing reads with poor quality. In this study, we employed two distinct variant callers to mitigate caller-specific bias in variant discovery, which significantly reduced the false discoveries resulting from the RT errors.

Furthermore, our RNA-SM discovery pipeline addressed the issue of alignment errors at splicing junctions by jointly utilizing two aligners, STAR and HISAT2, which employ distinct clipping strategies. In this way, potential bias from specific clipping strategies were effectively reduced. Moreover, aligners developed for RNA-seq data commonly utilize the soft clipping strategy to improve alignment efficiency for high-throughput analysis. This strategy often marks unmapped nucleotides at the 5’ or 3’ end of the read as “soft-clipped” regions due to various reasons, such as incompletely removed adapter sequences or short exonic junctions. However, mutation detectors, such as Mutect2, detect SMs from the entire RNA reads regardless of their locations (within aligned or soft-clipped regions). Thus, it is important to filter out soft-clipped regions in RNA-seq reads to ensure the accuracy of the results. Our analysis showed that false discoveries were associated with significantly lower read depth at the variant site compared to adjacent nucleotides. Based on this observation, we determined the read depth surrounding a variant site to exclude those with uneven read coverage. To alleviate the interference from RNA editing, our pipeline not only utilized existing RNA editing databases but also incorporated a machine learning approach to predict novel RNA editing sites. By employing both strategies, we were able to effectively minimize the RNA editing effects, resulting in more accurate detection of RNA-SMs than other available tools. Furthermore, the successful application of IMAPR to multiple independent validation datasets further demonstrated its broad applicability.

Currently, WXS is the primary method used for discovering tumor SMs. TCGA provides the WXS data from over 10,000 tumors for SM discovery across various cancer types. Our research has shown that high-coverage WGS can identify additional SMs that may not otherwise be detectable by standard WXS due to the read depth or GC content issues. However, in the TCGA database, high-coverage WGS data were presented for only about 2000 tumors. In contrast, over 10,000 tumors have associated RNA-seq data readily available for public access. Interestingly, our RNA-SM discovery pipeline can identify SMs that were missed by WXS analysis but confirmed by high-coverage WGS analysis. These novel RNA-SMs can complement previously reported WXS-based somatic mutations (**WXS-SMs**) to provide a more complete picture of the mutational profiles across various cancers in TCGA.

Importantly, novel mutations identified by RNA-SM analysis can provide a new perspective to guide patient-specific treatment. By utilizing these novel RNA-SMs, we could potentially improve treatment efficacy and offer more personalized therapeutic strategies. Using *KRAS* mutations as an example, prior studies have indicated that *KRAS* mutations are associated with more aggressive disease and poor response to various treatment^34^. Our findings suggest that patients with *KRAS* mutations that were exclusively identified through RNA-SM analysis have inferior survival outcomes. Functional analysis further revealed that *KRAS* mutations led to upregulation of WNT, MAPK, and IL-6/JAK/STAT signaling pathways, which are known to facilitate cancer cell proliferation, growth, and invasion, resulting in more aggressive disease and reduced treatment response^35–37^. Patients newly diagnosed with *KRAS* mutations could potentially benefit from personalized treatments that are tailored specifically to their genetic profiles. For example, in lung adenocarcinoma patients with *KRAS* mutations, applying treatments with MEK, ERK, or SHP2 inhibitors had better outcomes than standard treatment targeting *EGFR*^38^.

Besides *KRAS*, our RNA-SM analysis also identified novel mutations in *PIK3CA*, *NOTCH1*, and *ERBB2*, among many other genes. At present, drugs targeting these mutations have been developed or are being developed^39,40^. Thus, patients harboring these gene mutations could potentially benefit from targeted therapies. In particular, as for immunotherapy, previous reports have suggested a significant association between *CDKN2A* mutations and response to treatment^41^. In our study, our RNA-SM analysis also identified new patients with mutations in *CDKN2A*. Thus, these patients could benefit from immunotherapies such as neoantigen vaccines, adoptive T-cell therapy, oncolytic viruses, or combination approaches. In summary, comprehensive knowledge of gene mutations could facilitate the design of more precise and tailored therapeutic strategies to improve treatment and patient outcomes.

Moreover, besides the RNA-seq data hosted in TCGA, our pipeline can be broadly applied to tumor RNA-seq data for SM discovery. In particular, in the past two decades, a large quantity of RNA-seq datasets have been generated for gene expression studies, and they are readily accessible in public data repositories. These public datasets could be exploited for tumor SM discovery using our RNA-SM discovery pipeline. In summary, our findings demonstrate that combined analysis of RNA-SMs and DNA-SMs can depict a more comprehensive landscape of somatic mutations in cancer, which could potentially help develop individualized treatment plans for improved patient care.

## Methods

### Retrieval of DNA-seq data

Two types of DNA-seq data, WXS and WGS data, from TCGA were included in our study for DNA-SM discovery. WXS-SMs were obtained directly from the TCGA data portal (https://portal.gdc.cancer.gov/). These mutations were identified by TCGA using the somatic aggregation workflow as described in the data portal. As there is no WGS-based somatic mutation (**WGS-SM**) presented by TCGA, we followed the same TCGA DNA-seq analysis workflow to identify SMs using the WGS data. Raw WGS reads were downloaded from the TCGA data portal and aligned to the human reference genome GRch38.d1.vd1 using BWA^42^. Aligned WGS reads were then filtered to remove duplicates and recalibrated to exclude sequencing errors using the GATK toolkits^43^. Finally, these filtered reads were used for the discovery of DNA-SMs using Mutect2.

### Retrieval of RNA-seq data

Raw RNA-seq data were downloaded from the TCGA data portal. We included 32 major types of cancer with a total of 8048 cases that met the selection criteria: (1) available paired-end RNA-seq data from the tumor with a minimum of five million reads aligned to the human genome and (2) available WXS data from paired blood samples of the same patients. In our study, blood WXS data were used as a reference to identify and exclude germline mutations from the SM analysis.

### RNA-SM discovery pipeline

We developed the IMAPR pipeline to detect SMs from RNA-seq data. As shown in Fig. S1, this pipeline utilized an integrated strategy, incorporating two sequence aligners and two variant callers. In the first sequence alignment step, raw RNA-seq data were mapped to the human genome GRch38.d1.vd1 (https://gdc.cancer.gov/about-data/gdc-data-processing/gdc-reference-files) using STAR^44^, with specific parameters summarized in Supplementary Table S1. Mapped RNA-seq reads were then analyzed with Mutect2^11^ for variant calling. Next, for the second alignment step, variant-containing RNA-seq reads were extracted and aligned to the human genome GRch38.d1.vd1 using HISAT2^45^, with specific parameters summarized in Supplementary Table S1. HISAT2 was adopted as a second aligner as it has robust performance in excluding reads with mismatches or sequencing adapters^46^. Realigned reads were then subject to repeated variant calling using Mutect2. Mutect2 utilizes a more permissive algorithm to identify variants with low allele frequency, which could result in higher false positive rates^47^. To alleviate this concern, besides Mutec2, we further employed an independent approach for a second round of variant calling using the mpileup function in samtools^48^.

Next, we applied eighteen filters (see Supplementary Methods for details) to remove candidate variants that were likely to be false positives. The remaining candidate variants underwent an additional filter based on machine learning to remove variants that were likely caused by RNA editing. After passing all the aforementioned filters, the remaining variants were considered as RNA-SMs, which were further mapped to respective genes to predict their functional impact using ANNOVAR^49^ with GENCODE version 36 as Ref ^50^. It is important to note that, before each variant calling step using Mutect2, we applied GATK toolkits^43^ to preprocess aligned RNA-seq reads. This preprocess included removing duplicated reads and splitting reads that contained splicing junctions based on identified splicing sites; additionally, base recalibration was performed on all reads to remove low-quality variants resulting from sequencing errors. This preprocessing step is important for improving the accuracy of variant calling by reducing false positive predictions.

To develop a machine learning-based model for removing RNA-level variants (e.g., from RNA editing), we used the SMs detected from 45 TCGA LUADs as the training dataset. For model validation, we used an independent set of SMs identified from 20 TCGA LUSCs and 35 HNSCs. Of note, DNA-SMs identified from a combined dataset of WXS and WGS were used as a reference to label the RNA-SMs. As shown in Fig. S2c, we extracted 26 mutational features for each SM (details of these filters are presented in Supplementary Methods). Five machine learning models, including random forest, logistic regression, multiplayer perceptron, XGBoost, and support vector machine, were applied to identify false discoveries. The hyper-parameters for each learning method were optimized by 5-fold cross-validation.

### Correlative analysis of RNA-SMs and DNA-SMs

We performed a per-patient correlation analysis between RNA-SMs and two types of DNA-SMs (WXS-SMs and WGS-SMs). WGS covers the entire genome, whereas WXS focuses on the protein-coding regions of the genome. To address variations related to coverage differences across sequencing platforms, in the correlative analysis, we only compared the SMs that were sufficiently covered in both RNA- and DNA-seq data. Specifically, SMs from sites with <10 reads in any sequencing dataset were excluded from the comparison analysis.

### Analysis of significantly mutated genes

To identify SMGs, we utilized dNdScv^51^, which employs the maximum likelihood dN/dS method to estimate the global prevalence of positive and negative selection in cancer and somatic evolution. dNdScv was developed to identify SMGs using DNA-seq data; however, in RNA-seq analysis, genes with low or no expression may have limited or no reads, resulting in insufficient coverage for accurately detecting somatic mutations. Thus, RNA-seq data may not capture mutations in these genes. As a result, dNdScv could misidentify such genes as undergoing negative selection. To address this issue, we set a cut-off to exclude lowly expressed genes with an average TPM (transcripts per million) <1 in a specific cancer type. The analysis for the SMGs was performed based on the human genome GRch38.d1.vd1, and the SMGs were determined based on an adjusted *p* value <0.05. Finally, All SMGs were visualized using the Oncoplot R package^52^.

### Analysis of mutational signatures

We utilized SignatureAnalyzer^21^ to predict mutational signatures based on identified SMs. Mutational signatures (version 3.3) in the COSMIC^25^ were used as reference to determine potential etiology of the SMs. Each SM was associated with a signature if its likelihood of association to the signature was greater than 0.75. Based on the results of non-negative matrix factorization, we allocated mutational signatures to specific cancer types.

### Gene set variation analysis

Gene expression data were compiled from the OncoDB database^53^. The hallmark gene sets were downloaded from the MSigDB database^54^. The GSVA R package^55^ were employed to compute pathway enrichment scores.

### Statistics and reproducibility

Statistical significance was assessed by calculating *p* values using various statistical methods. A *p* value <0.05 was considered statistically significant. Cross-platform comparison for sequencing coverage was performed with Student’s *t*-test. Additionally, we employed Student’s *t*-test to compare the enrichment scores between patients with or without RNA-SMs. Further, Correlation of cross-platform mutation counts was performed with Pearson correlation analysis.

### Reporting summary

Further information on research design is available in the Nature Portfolio Reporting Summary linked to this article.

### Supplementary information

Supplementary Information

Description of Additional Supplementary Files

Supplementary Data

Reporting Summary

## Data Availability

Based on identified RNA-SMs, we constructed a pan-cancer SM atlas for 32 types of cancer and published it on the OncoDB website (https://oncodb.org). The atlas integratively presents the SMs identified from both DNA-seq and RNA-seq data. A detailed online tutorial has been provided to help user to visualize SMs and correlate SMs with clinical data. In addition, users can retrieve all identified SMs via the data download interface. The source data behind the graphs in the paper can be found in Supplementary Data.

## Code availability

All source code and instruction for the IMAPR pipeline is available through GitHub (https://github.com/wang-lab/IMAPR), Zenodo (10.5281/zenodo.11175442), and Docker (https://hub.docker.com/r/wanglabbioinfo/imapr).
