# Supplementary material for: Pan-cancer discovery of somatic mutations from RNA sequencing data
Source: Commun Biol. 2024 May 23;7:619. doi: 10.1038/s42003-024-06326-y (PMC11116503; doi:10.1038/s42003-024-06326-y)
Supplement: Supplementary file 1 — Supplementary Information [file 42003_2024_6326_MOESM1_ESM.pdf]

# Supplementary Information for

# Pan-Cancer Discovery of Somatic Mutations from RNA-Seq Data

Gongyu Tang [1,2], Xinyi Liu[1], Minsu Cho[1], Yuanxiang li[1], Dan-Ho Tran[1], and Xiaowei Wang [1,3,*]

[1]Department of Pharmacology and Regenerative Medicine, University of Illinois at Chicago, Chicago, IL, USA. [2]Department of Mechanical Engineering and Materials Science, Washington University in St. Louis, St. Louis, MO, USA. [3]University of Illinois Cancer Center, Chicago, IL, USA.

**This PDF file includes:**

Supplementary Methods
Supplementary Table S1
Supplementary Figures S1 to S11
Supplementary References

# Supplementary File

# Contents:

**Supplementary Methods**


1. Mutation filters implemented in the IMAPR pipeline

2. Mutational features in the stacking learning model


**Supplementary Table**


Table S1. Summary of alignment parameters


**Supplementary Figures**


Fig. S1. Workflow of the IMAPR pipeline.

Fig. S2. The stacking model included in IMAPR.

Fig. S3. Validation of IMAPR performance.

Fig. S4. The SMGs in cervical cancer, as identified with the DNA-SMs using dNdScv.

Fig. S5. The SMGs in cervical cancer, as identified with a combined set of RNA-SMs and DNA-SMs using dNdScv.

Fig. S6. Counts of the DNA-SMs in PIK3CA across different protein domains. The positions of the amino acids are displayed along the x-axis.

Fig. S7. Mutational signatures in cervical cancer, as identified with the DNA-SM data.

Fig. S8. Pan-cancer somatic mutation profiles as revealed by combined RNA-seq and DNA-seq data.

Fig. S9. Pan-cancer somatic mutation profiles for top-ranking oncogenes / tumor suppressors as revealed by combined RNA-seq and DNA-seq analysis.

Fig. S10. Percentage of the tumors that harbor mutations in top-ranking genes across 32 cancer types, as revealed by combined RNA-SM and DNA-SM analysis.

Fig. S11. Pan-cancer mutational signatures discovered using combined RNA-SMs and DNA-SMs across 32 cancer types.

## Supplementary Methods

### 1. Mutation filters applied in the IMAPR pipeline

(1) Dual alignment filter.

We used two aligners, STAR and HISAT2, in IMAPR to align RNA-seq reads. Subsequently, we identified somatic mutations (SMs) with the aligned RNA-seq reads from two aligners. To reduce false positives, we excluded candidate SMs discovered only by a single aligner in IMAPR.

(2) Dual variant calling filter.

We used two variant calling algorithms, Mutect2 and samtools, in IMAPR to detect variants. To reduce false positive SMs, we excluded candidate SMs discovered only by a single variant caller in IMAPR.

(3) Gene filter.

We only included candidate SMs identified in genic regions and excluded candidate SMs identified in pseudogenes. Moreover, we excluded candidate SMs identified in immunoglobulin or HLA genes.

(4) Panel of normal (PON) filter.

To remove any germline variants that might be mistakenly identified as SMs, we excluded candidate SMs found in the panel of normal (PON) file. Specifically, we used the "MuTect2.PON.5210.vcf" file as the PON reference, which was obtained from the TCGA data portal (https://gdc.cancer.gov/about-data/gdc-data-processing/gdc-reference-files).

(5) RNA-edits filter.

RNA editing is a post-transcriptional process that modifies the nucleotide sequence of RNA molecules, which can be misidentified as SMs. To remove known RNA-editing events during

SM discovery, we used three RNA-edit databases [REDIportal [1], RADAR [2], DARNED [3]] to merge reported RNA editing events. Any candidate somatic mutations found in the combined RNA-edit database were excluded.

(6) Long edits filter.

Candidate SMs longer than five nucleotides were excluded, as longer insertions/deletions in short reads were mainly the result of splicing events at the transcriptional level.

(7) Mapping quality filter.

Candidate SMs with an average mapping quality score less than 50 were excluded.

(8) Variants position filter.

Candidate SMs with an average distance of less than 6 nucleotides between variant positions and read boundaries were excluded.

(9) Sequencing quality filter.

We conducted Wilcoxon rank-sum test to compare the sequencing quality scores of base calls supporting the alternate allele versus those supporting the reference allele. Candidate SMs with a p-value <0.05 and a $\log_2$ fold change <-0.5 were excluded. Sequencing quality scores were extracted using the mpileup function in samtools.

(10) Short tandem repeats.

To reduce false positive SMs caused by inherent instability of short tandem repeat (STR) regions, we excluded any candidate SM found within these STR regions, which were identified by Mutect2. In addition, we excluded candidate SMs that fall into single nucleotide repeat (n>4) regions as sequencing inaccuracy was frequently observed in these regions.

(11) Multiallelic filter.

Candidate SMs containing multiple types of nucleotide change at the same position were excluded in IMAPR.

(12) Supporting reads filter.

We filtered out candidate SMs with fewer than three supporting reads and a variant allele frequency (VAF) lower than 0.1.

(13) High frequency filter.

We excluded candidate SMs with a variant allele frequency (VAF) greater than 0.9, as we observed a significant enrichment of such variants in false postives. This may be attributed to various factors such as allele-specific expression, RNA editing, or sequencing artifacts.

(14) Germline variants filter.

We excluded any candidate SM identified in matched normal samples.

(15) TLOD score filter.

TLOD score is calculated by Mutect2 using maximum likelihood approach by comparing VAF between RNA-seq data and background sequencing errors. We excluded candidate SMs with TLOD score less than 5.6.

(16) Coverage filter.

Our analysis showed that falsely discovered SMs had significantly lower read depth compared to adjacent nucleotides. Based on this observation, we conducted a chi-square uniform test of sequencing coverage on each side of the SMs. Candidate SMs with a p-value <0.05 and fold change >1.5 were excluded.

(17) Cluster events filter.

We excluded candidate SMs co-occurring in the same local assembly interval as revealed by Mutect2. These candidate SMs were mostly the result of noisy alignment.

(18) Blacklist region filter.

We excluded candidate SMs discovered on the sex chromosomes and unfinished chromosomal scaffolds due to their high potential for false-positive calls.

**2. Mutational features included in the stacking model**

(1) Read depth of the variant site in the tumor sample.

(2) Read count supporting the variant in the tumor sample as identified by Mutect2.

(3) Variant frequency in the tumor sample as identified by Mutect2.

(4) Average mapping quality of the reads supporting the variant in the tumor sample.

(5) Average distance between the variant positions and read boundaries in the tumor sample.

(6) Log likelihood score of the variant being a somatic mutation versus being a germline mutation.

(7) Log likelihood score of the variant being present in the normal sample versus being an artifact.

(8) Log likelihood score of the variant being present in the tumor sample versus being an artifact.

(9) Prior odds of the variant being a somatic mutation based on reference population frequency.

(10) Median sequencing quality of all reads supporting the variant in the tumor sample.

(11) Median sequencing quality for all reads supporting non-variant calls in the tumor sample.

(12) Log2 fold change of the median of sequencing quality between the variant reads and non-variant reads in the tumor sample.

(13) P-value from Wilcoxon rank sum test of the sequencing quality between the variant reads and non-variant reads in the tumor sample.

(14) Adjacent read depths surrounding the variant site.

(15) P-value from chi-square uniform distribution test of the read depth distribution in regions adjacent to the variant site.

(16) Read count supporting the variant in the tumor sample as identified by samtools mpileup.

(17) Variant frequency in the tumor sample as identified by samtools mpileup.

(18) Variant distance bias score based on the distance between the variant position and read boundary.

(19) Symmetric odds ratio bias score based on sequencing direction bias in reads supporting the variant.

(20) Read position bias score based on the position of the variant within the reads.

(21) Mapping quality bias score based on the mapping quality of the variant reads vs. non-variant reads.

(22) Mapping quality symmetric bias score based on the sequencing direction bias in reads supporting the variant.

(23) Sequencing quality bias score based on the sequencing quality of the variant reads vs. non-variant reads.

(24) Fraction of variant reads with mapping quality score of zero.

(25) Categories of nucleotide alteration.

(26) Categories of genomic location.

**Supplementary Table**

**Table S1. Summary of alignment parameters**

| STAR alignment parameters | |
|---|---|
| STAR | |
| --alignIntronMax | 1000000 |
| --alignIntronMin | 20 |
| --alignMatesGapMax | 1000000 |
| --alignSJDBoverhangMin | 1 |
| --alignSJoverhangMin | 8 |
| --alignSoftClipAtReferenceEnds | Yes |
| --chimJunctionOverhangMin | 15 |
| --chimMainSegmentMultNmax | 1 |
| --chimOutJunctionFormat | 1 |
| --chimSegmentMin | 15 |
| --limitSjdbInsertNsj | 1200000 |
| --outFilterIntronMotifs | None |
| --outFilterMatchNminOverLread | 0.33 |
| --outFilterMismatchNmax | 999 |
| --outFilterMismatchNoverLmax | 0.1 |
| --outFilterMultimapNmax | 20 |
| --outFilterScoreMinOverLread | 0.33 |
| --twopassMode | Basic |
| --outSAMmapqUnique | 60 |

| Hisat2 alignment parameters | |
|---|---|
| Hisat2 | -min-intronlen |
| -max-intronlen | 500000 |
| -k | 5 |
| -I | 0 |
| -x | 500 |

**Supplementary References**


1. Picardi E, D'Erchia AM, Lo Giudice C, Pesole G: REDIportal: a comprehensive database of A-to-I RNA editing events in humans. *Nucleic Acids Res* 2017, 45:D750-D757.
2. Ramaswami G, Li JB: RADAR: a rigorously annotated database of A-to-I RNA editing. *Nucleic Acids Res* 2014, 42:D109–D113.
3. Kiran A, Baranov PV: DARNED: a DAtabase of RNa EDiting in humans. *Bioinformatics* 2010, 26:1772–1776.

# Fig. S1

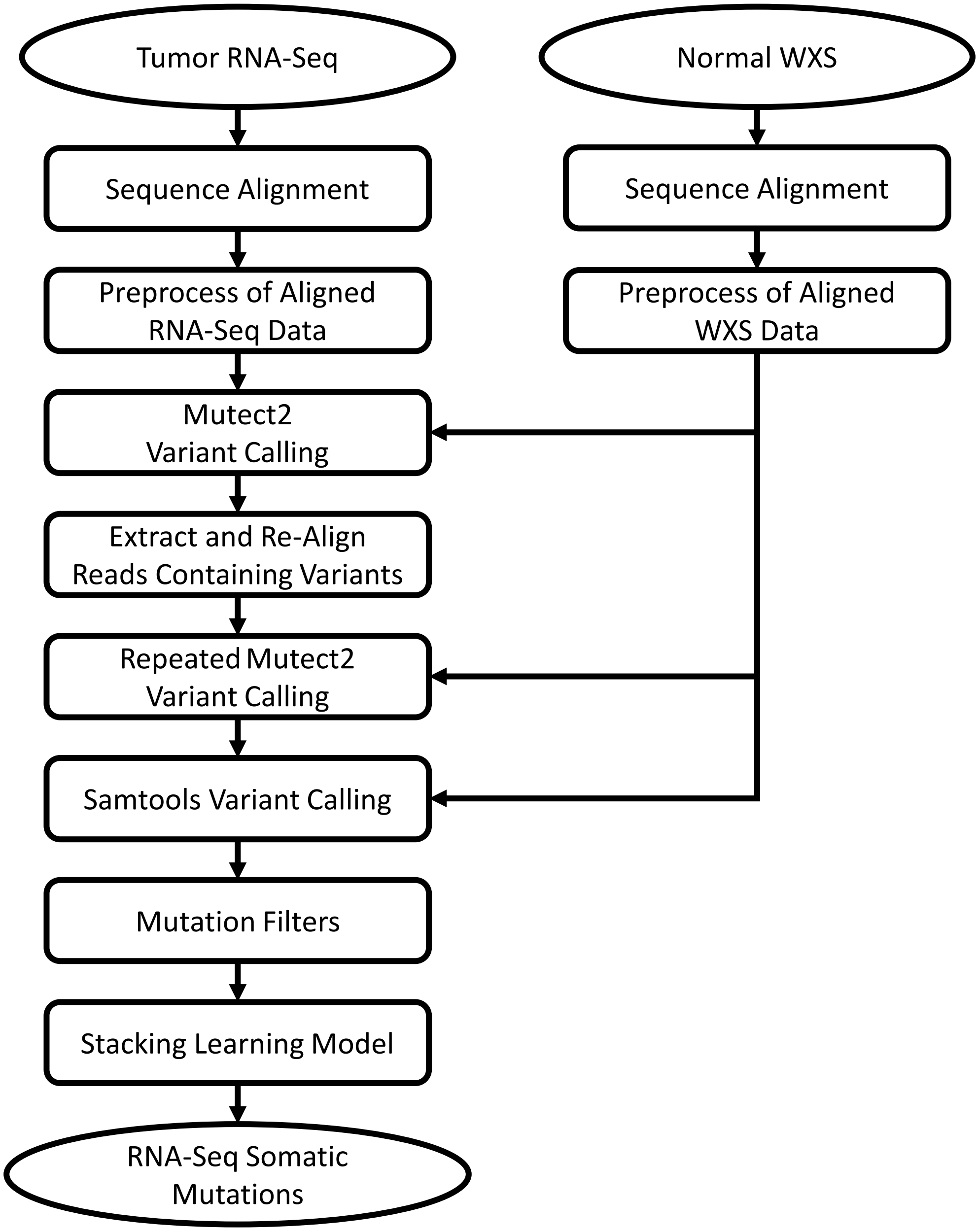


**Supplementary Fig. S1.** Workflow of the IMAPR pipeline.

## Fig. S2

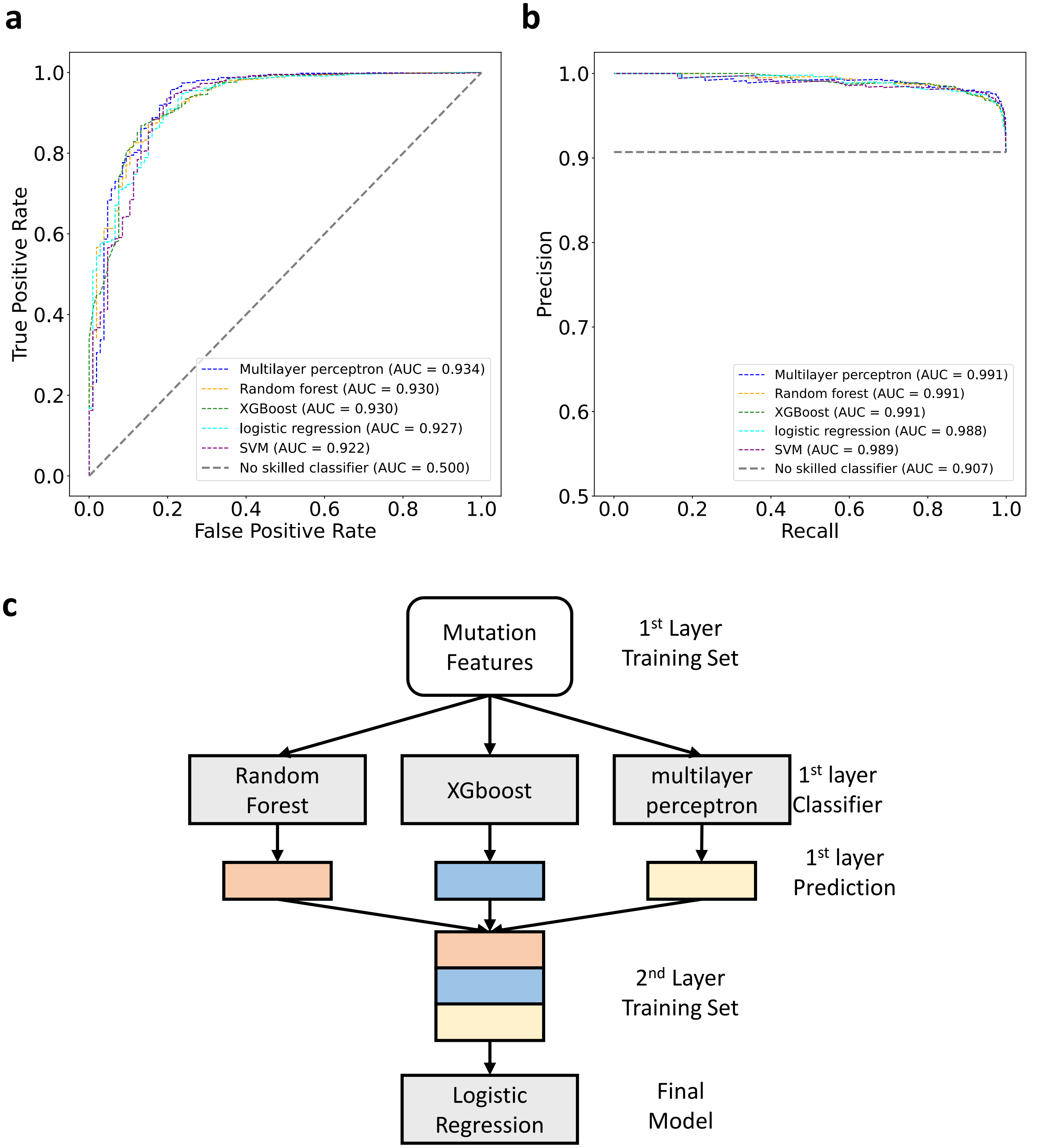


**Supplementary Fig. S2.** The stacking model included in IMAPR. The training data were used to build individual machine learning models, and the performance of these models was assessed by the AUC values of ROC (**a**) or PR curve (**b**) analysis using the WGS data as reference. (**c**) Workflow of the stacking model.

Fig. S3

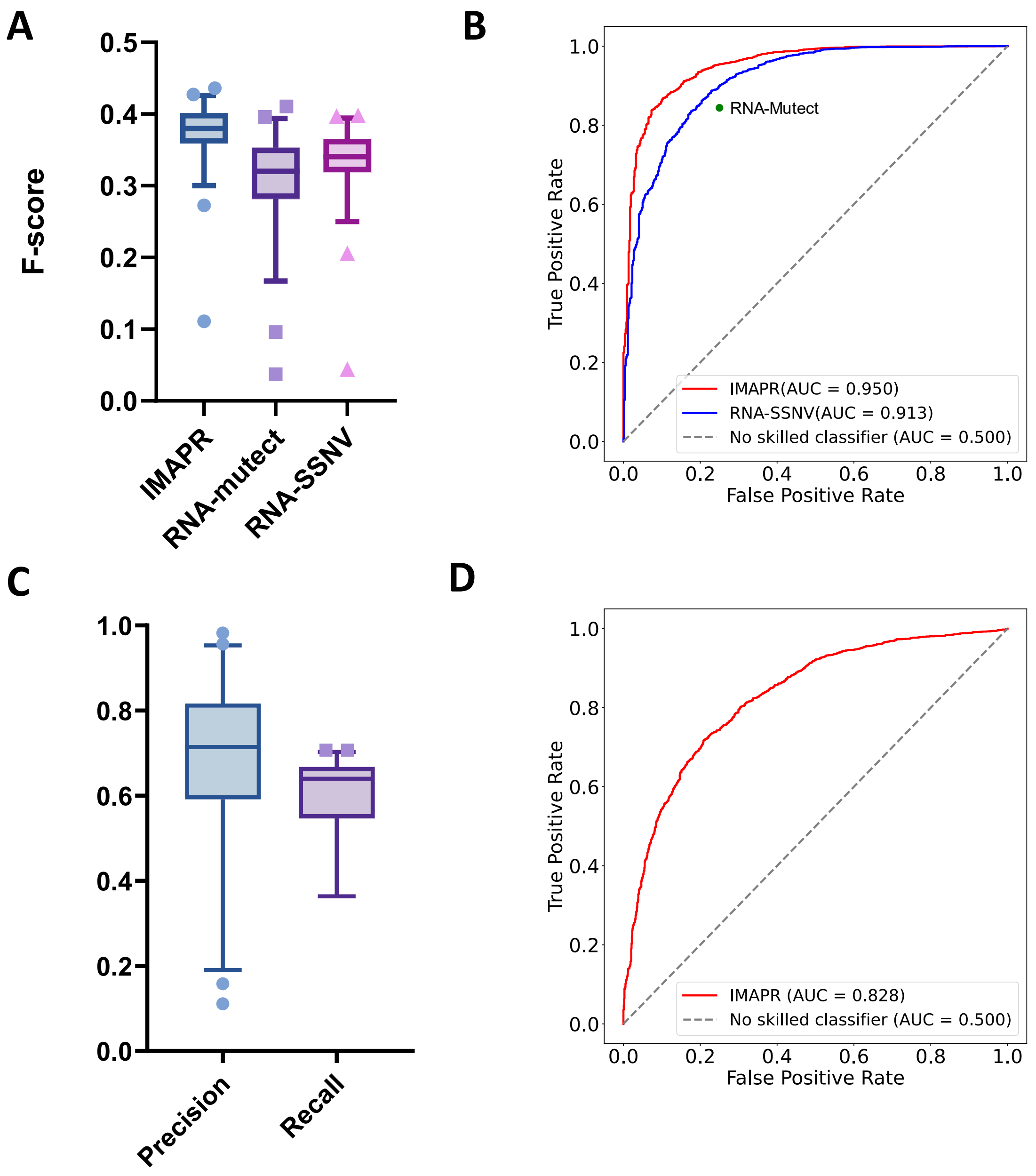


**Supplementary Fig. S3.** Validation of IMAPR performance. **(a)** F-score distribution of the RNA-SMs across individual tumors as discovered by IMAPR, RNA-Mutect or RNA-SSNV. **(b)** The performance of IMAPR, RNA-Mutect and RNA-SSNV were assessed by the AUC-ROC values using TCGA WGS data as reference. **(c)** Precision and recall distribution of the RNA-SMs across individual tumors as discovered by IMAPR on the Mun dataset **(d)** The performance of IMAPR was assessed by the AUC-ROC values on the Mun dataset.

# Fig. S4

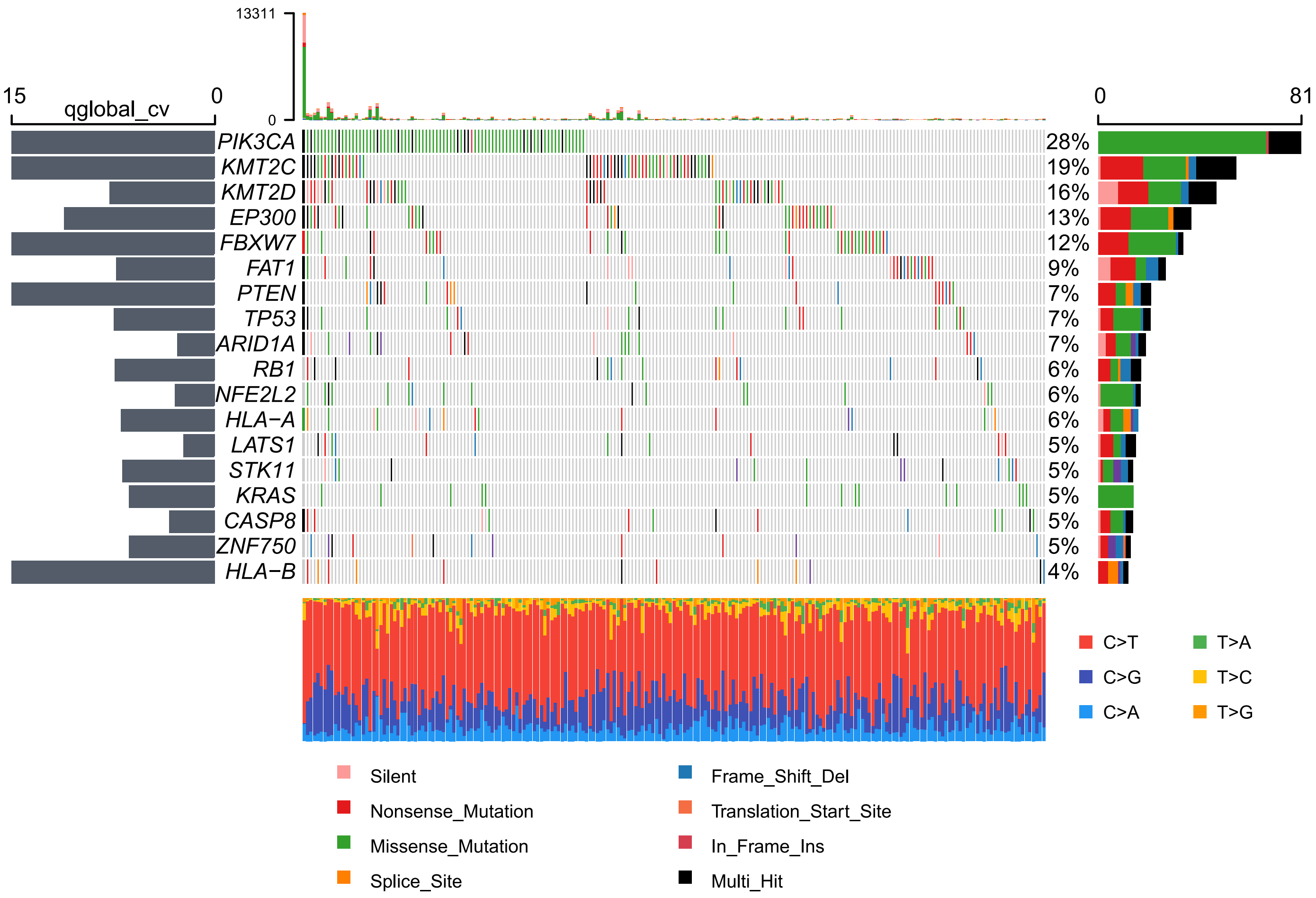


**Supplementary Fig. S4.** The SMGs in cervical cancer, as identified with the DNA-SMs using dNdScv.

# Fig. S5

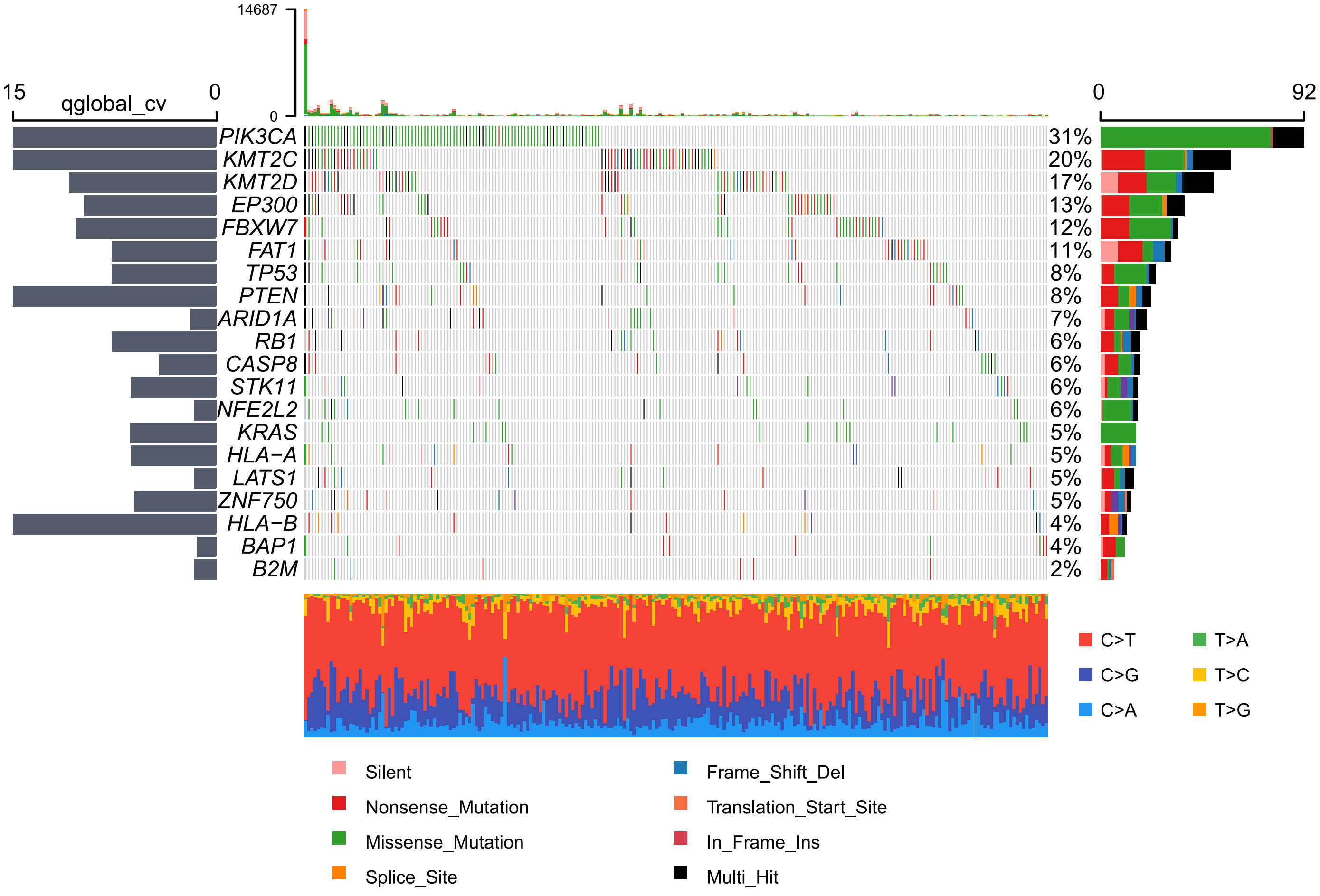


**Supplementary Fig. S5** The SMGs in cervical cancer, as identified with a combined set of RNA-SMs and DNA-SMs using dNdScv.

**Fig. S6**

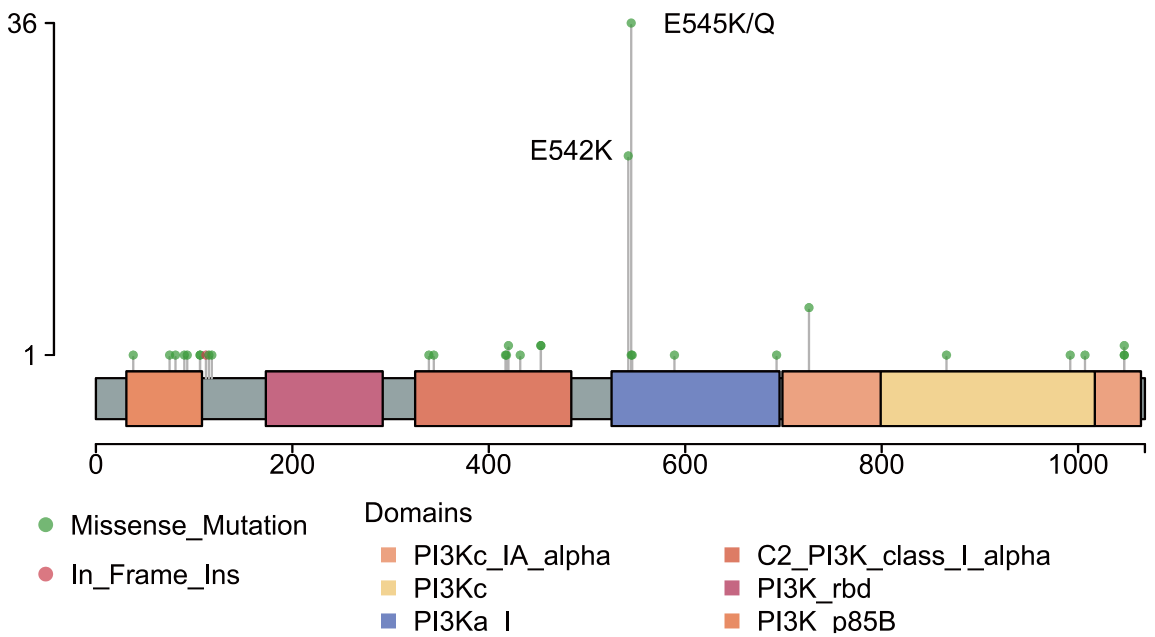


**Supplementary Fig. S6** Counts of the DNA-SMs in PIK3CA across different protein domains. The positions of the amino acids are displayed along the x-axis.

**Fig. S7**

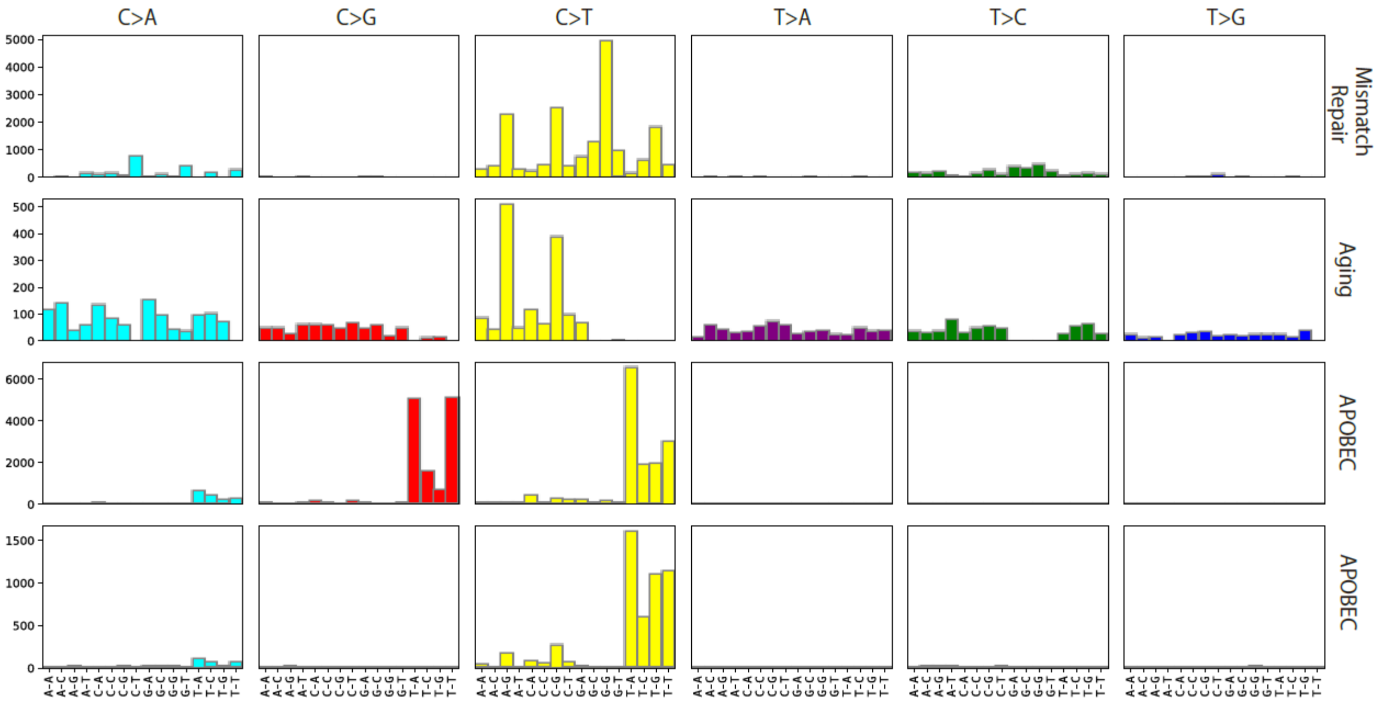


**Supplementary Fig. S7.** Mutational signatures in cervical cancer, as identified with the DNA-SM data. These included DNA mismatch repair, APOBEC, and Aging.

# Fig. S8

**a**

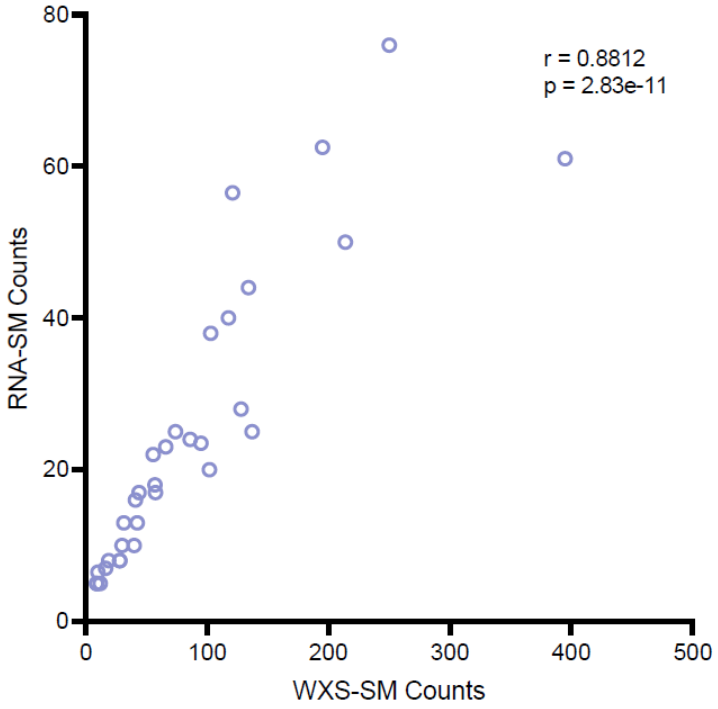


**b**

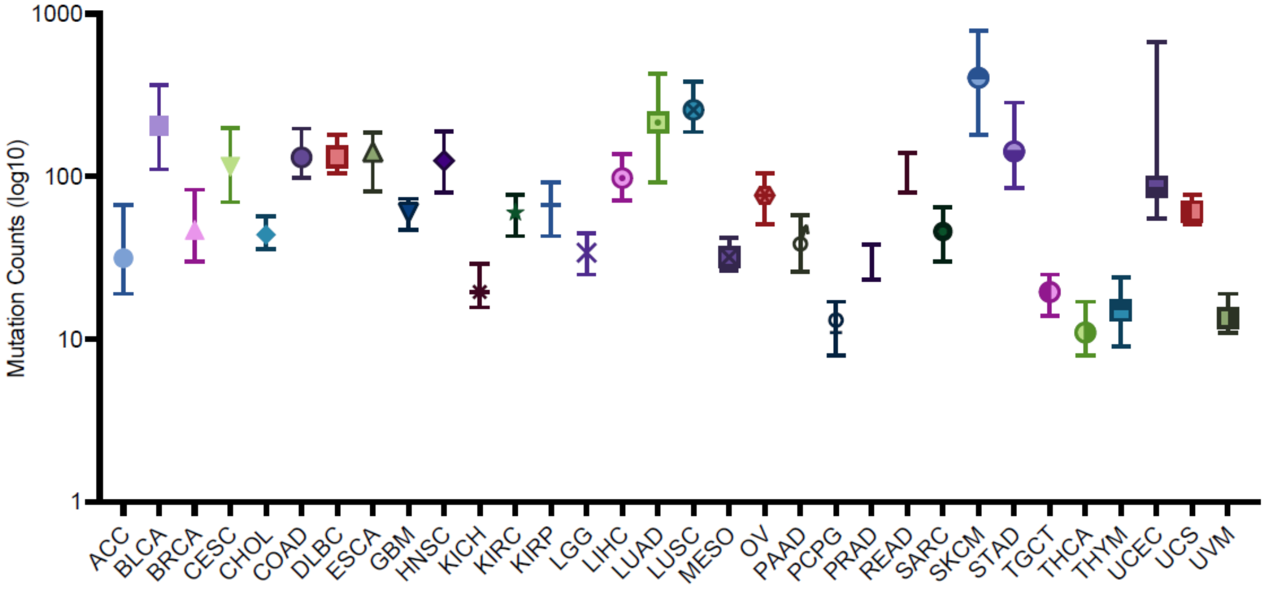


**Supplementary Fig. S8.** Pan-cancer somatic mutation profiles as revealed by combined RNA-seq and DNA-seq data. (**a**) Correlation between the RNA-SM and DNA-SM counts across 32 cancer types. (**b**) Distribution of the mutation counts in individual patients across 32 cancer types using a combined set of DNA-SMs and RNA-SMs.

# Fig. S9

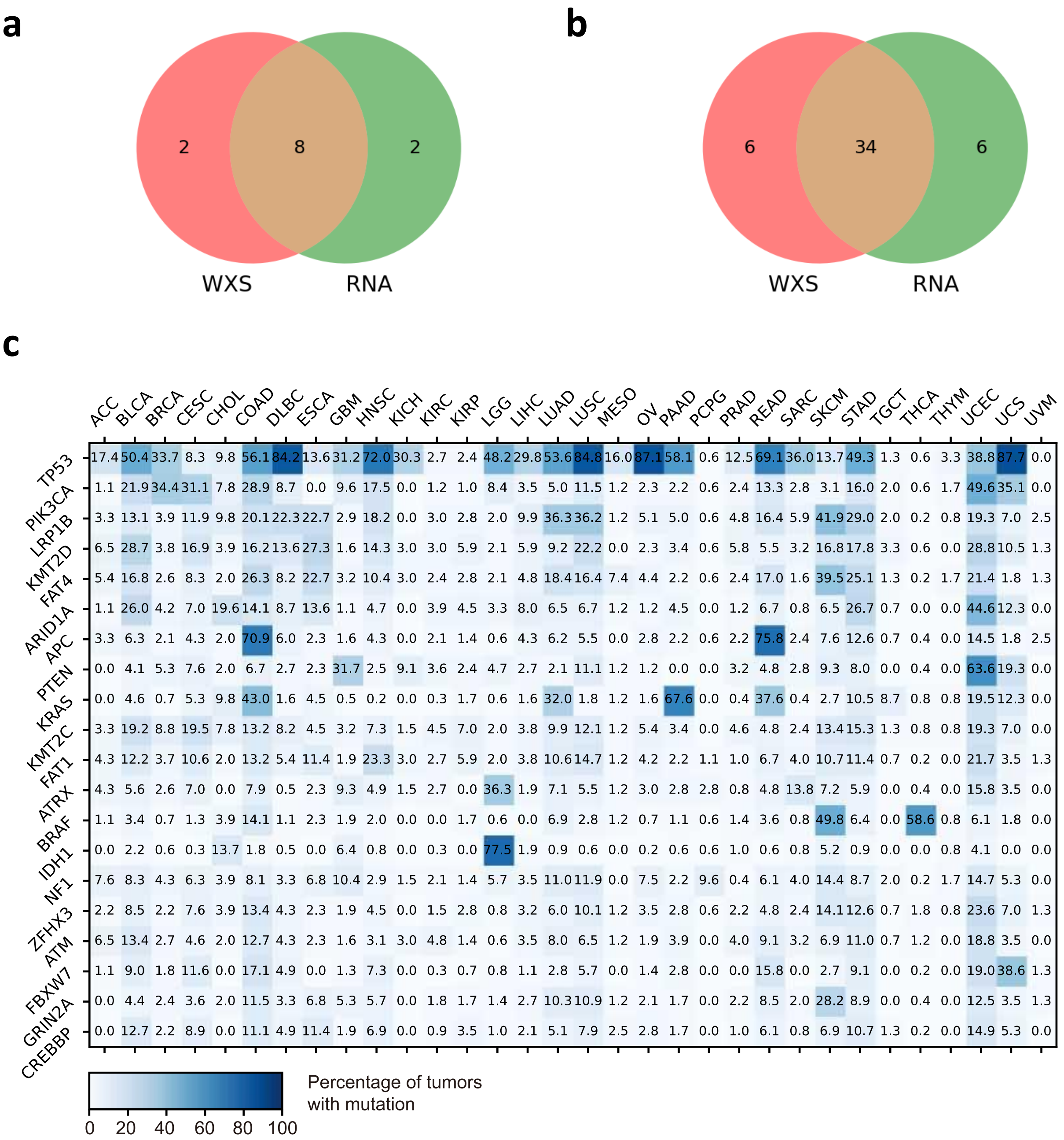


**Supplementary Fig. S9.** Pan-cancer somatic mutation profiles for top-ranking oncogenes / tumor suppressors as revealed by combined RNA-seq and DNA-seq analysis. (**a**) Venn diagram of top ten most mutated genes as determined by RNA-seq and DNA-seq analyses, respectively. (**b**) Venn diagram of top 40 most mutated genes. (**c**) Percentages of the tumors that harbor mutations in top-ranking oncogenes / tumor suppressors across 32 cancer types, as revealed by combined RNA-SM and DNA-SM analysis. Twenty most mutated genes are presented.

# Fig. S10

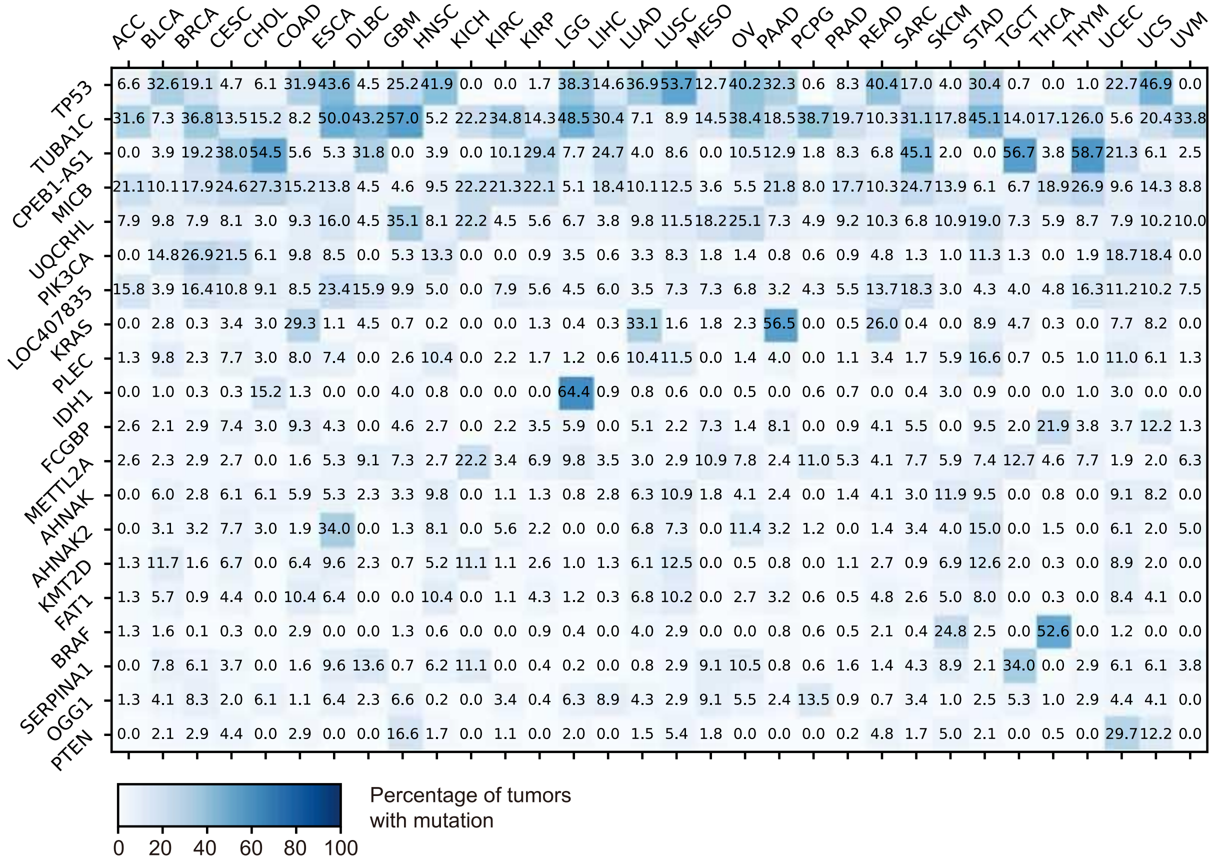


**Supplementary Fig. S10.** Percentage of the tumors that harbor mutations in top-ranking genes across 32 cancer types, as revealed by combined RNA-SM and DNA-SM analysis. Twenty most mutated genes are presented.

## Fig. S11

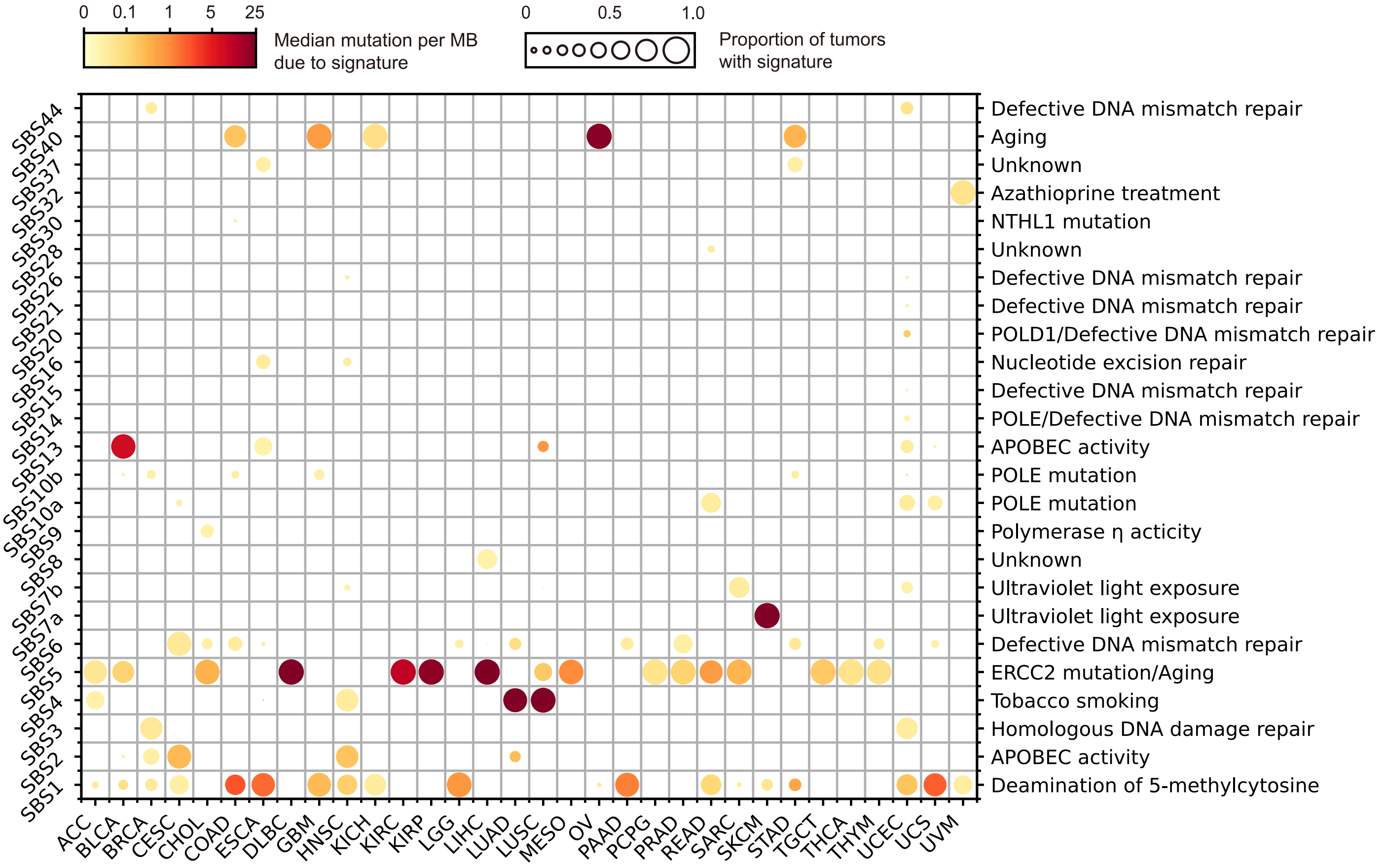


**Supplementary Fig. S11.** Pan-cancer mutational signatures discovered using combined RNA-SMs and DNA-SMs across 32 cancer types. The size of each dot represents the proportion of tumors with the mutational signature in each cancer type. The color of each dot represents median mutational burden in all individual tumors of the same type. Only tumors with identified mutational signature were included in the analysis.
