# Supplementary material for: Pan-cancer discovery of somatic mutations from RNA sequencing data
Source: Commun Biol. 2024 May 23;7:619. doi: 10.1038/s42003-024-06326-y (PMC11116503; doi:10.1038/s42003-024-06326-y)
Supplement: Supplementary file 2 — Description of Additional Supplementary Files [file 42003_2024_6326_MOESM2_ESM.pdf]

Description of Additional Supplementary Files

File name: Supplementary Data
Description: The source data for the graphs in the paper
